# Optimizing a rapid tissue culture method for steviol glycoside production from *Stevia rebaudiana* to address Egypt’s sugar deficit

**DOI:** 10.1038/s41598-025-10491-3

**Published:** 2025-07-15

**Authors:** Mostafa B. Abouelela, Mohamed Eid, Fekria M. Ali, Asmaa I. Owis

**Affiliations:** 1 Department of Pharmacognosy, College of Pharmacy, Egyptian Russian University, Badr City, 11829 Cairo Egypt; 2 Faculty of Organic Agriculture, Heliopolis University, Cairo, Egypt; 3 Department of Biotechnology, Faculty of Organic Agriculture, Heliopolis University, Cairo, Egypt; 4 Department of Pharmacognosy, Faculty of Pharmacy, Heliopolis University, Cairo, Egypt; 5 Department of Pharmacognosy, Faculty of Pharmacy, Beni-suef University, Beni-suef, Egypt

**Keywords:** Callus, Direct micropropagation, Indirect micropropagation, Multivariate data analysis, Plant tissue culture, Stevia rebaudiana, UPLC-MS/MS, Biotechnology, Plant sciences

## Abstract

Egypt has encountered a significant sugar scarcity since 2023, due to a water crisis, which has reduced local sugar production, prompting a search for alternative sources. *Stevia rebaudiana* (Asteraceae) is a natural source of steviol glycosides, which are high-intensity, low-calorie sweeteners with increasing demand in food and pharmaceutical industries. Despite its potential as a water-efficient alternative to sugar crops, Egypt lacks optimized protocols for stevia propagation and secondary metabolite enhancement. This study aimed to develop an efficient in vitro regeneration system for a local stevia genotype using callus induction, as well as both direct and indirect micropropagation and to assess its impact on steviol glycoside accumulation by comparison with conventionally soil-grown plant. Explants were cultured on Murashige and Skoog (MS) media supplemented with varying concentrations of BAP, NAA, and kinetin to evaluate callus formation, shoot proliferation, and root development. Optimal conditions yielded significantly higher shoot regeneration frequencies (up to 93%) and shoot number per explant (up to 12.6). Regenerated plants were acclimatized with a survival rate exceeding 85%. Ultra-Performance Liquid Chromatography-Tandem Mass Spectrometry (UPLC-MS/MS) was used for metabolite profiling of the four studied stevia. A total of 18 compounds were detected across the four studied stevia samples, including 11 phenolic compounds, and 7 diterpenoids, primarily stevioside, rebaudioside A, and rebaudioside C. Metabolite quantification based on relative peak areas revealed that the direct micropropagation strategy yielded the highest levels of stevioside and rebaudioside A (13.17 and 5.71%, respectively), surpassing those in soil-grown plants, callus-derived and indirectly propagated samples. Multivariate data analysis was conducted to identify relationships among metabolite markers in the four studied stevia samples. The metabolite profiles of both soil-grown and regenerated through direct micropropagation stevia was found to be similar, with both being rich in steviol glycosides. Notably, the growth duration varied among the four studied stevia. The soil-grown and indirectly micropropagated stevia took 180 and 196 days to reach maturity, respectively while stevia regenerated via direct micropropagation took 140 days, demonstrating a more rapid development. These findings demonstrated that direct micropropagation not only enhances growth but also conserves metabolic integrity, and highlights it as an ideal strategy for scalable production of sweetener under resource-restricted settings in arid and semi-arid regions.

## Introduction

Climate change poses a significant threat to the agricultural sector and the stability of food security in Egypt due to rising temperatures, shifting precipitation patterns, and increasing water scarcity^1^. According to UNICEF report, Egypt is facing an annual water deficit of around seven billion cubic meters and the country could run out of water by 2025, when it is estimated that 1.8 billion people worldwide will live in absolute water scarcity. With more than 85% of its freshwater supply originating from the Nile River, Egypt faces critical shortages due to population growth, upstream water management policies, and climate change^2^. Sugar is a crucial to Egypt’s economy, agriculture, and food security which mainly is derived from sugarcane and beet^3^. According to the United States Department of Agriculture (USDA), Egypt’s sugar production for the Marketing Year (2024/25) is decreased by 110,000 tons compared to (2023/24)^4^. Sugarcane, predominantly grown in Upper Egypt, is a highly water-intensive crop, consuming over 10,000–12,000 m^2^/ha annually (FAO, 2020). In contrast, sugar beet is cultivated in northern Egypt and consumes approximately 6000–7000 m^2^/ha, yet still imposes significant pressure on limited water resources. As a result, national strategies now emphasize crop diversification and the use of less water-demanding crop with higher-value secondary metabolites, such as *Stevia*, which requires considerably less irrigation (approximately 3000–4000 m^2^/ha) while offering a valuable alternative as a natural sweetener^5^.The genus *Stevia*, one of the most important in the Asteraceae family, comprises about 230 species with various pharmacological activities^6^. Among these species, *Stevia rebaudiana* (Bert.) Bertoni is noted for its sweetness^7^that discovered by Moises Santiago Bertoni in 1877 in Paraguay^8^. It is native to South America particularly Paraguay and Brazil, but now it is grown in different regions around the world, including Asia, Europe, and North America, owing to its importance in food and pharmaceutical industries as a natural substitute for sugar^7^. It is known as honey leaf, sweet leaf, and sweet herb due to its steviol glycosides, which are 100 to 300 times sweeter than sucrose without causing dental caries^8^. These compounds have gained international regulatory approval for use as natural sweeteners in over 60 countries, including approval by the FAO/WHO Joint Expert Committee on Food Additives (JECFA), the European Food Safety Authority (EFSA), and the U.S. FDA^9^. More than 30 steviol glycosides have been reported from stevia including stevioside, steviolbioside, rebaudioside A, B, C, D, E, F, and dulcoside^10^. Among the steviol glycosides, stevioside and rebaudioside A are the primary sweeteners found in stevia leaves^11^. Moreover, it contains a broad spectrum of active constituents belonging to several classes such as phenolic acids (e.g. caffeic acid, and quinic acid derivatives), and flavonoids (e.g. quercetin, and kaempferol glycosides)^12^. Numerous biological activities of stevia have been reported, including anti-caries, antimicrobial, antioxidant, anti-diabetic, anti-obesity, anti-hypertensive, and anti-tumor^10^. Moreover, stevia’s natural origin appeals to health-conscious consumers seeking to avoid artificial sweeteners, that could play a role in addressing the global issue of sugar-related health problems^13^. Since 1994, Stevia was registered and used as a safe natural sweetener in as soft drinks, baked goods, dairy products and in herbal teas in Egypt^14^. The biosynthesis of steviol glycosides involves complex enzymatic pathways that can be influenced by genotype, culture conditions, and propagation method. Ceunen and Geuns (2013)^15^ highlighted the chemical diversity of these glycosides and their biosynthetic regulation, while Lemus-Mondaca et al. (2012)^16^ emphasized the influence of environmental and cultivation factors on glycoside composition. In Egypt, where sugar imports remain high and freshwater resources are limited, and *S. rebaudiana* represents a strategic alternative to traditional sugar crops like sugarcane and sugar beet, the development of efficient micropropagation systems tailored to Egyptian agro-environments remains underexplored. Our study addresses this gap by optimizing in vitro regeneration methods for locally cultivated stevia genotype, with the dual goal of enhancing both plant biomass and steviol glycoside content. This subsequently will support the large-scale cultivation of *S. rebaudiana* as a sustainable raw material for natural sweetener industry which is aligned with Egyptian national strategies focusing on reduced water consumption and sugar imports. The regulatory guidelines established by the Egyptian Organization for Standardization and Quality (EOS) encourage the expansion of stevia usage as an alternative sweetener to conventional sugars^17^. However, the traditional way of stevia cultivation in Egypt faces several challenges, prompting the exploration of more efficient alternative propagation method^18^. Propagation techniques for stevia includes both sexual (seeds) and asexual strategies which encompass vegetative (stem cuttings) and biotechnological (in vitro) cultures. Sexual propagation is often hindered by several obstacles such as low seed germination rates and prolonged harvest times. Asexual propagation by stem cuttings may improve the uniformity but is relying on ecological environments and is not appropriate for large-scale production^19^. In vitro culture is a valuable technique used for the cultivation of plants with precious secondary metabolites, and for their large-scale production within a short period of time^20^. It encompasses two primary approaches, the first one is called direct micropropagation, which is a rapid method of plant generation that develops shoots and roots directly^21^. The other one is called indirect micropropagation, which takes longer due to the formation of an intermediate tissue called the callus^21^. Micropropagation techniques have been instrumental in enhancing the production of secondary metabolites in medicinal plants. By manipulating in vitro culture conditions and introducing specific elicitors, the type of explants used, the type and concentration of plant growth regulators, and environmental conditions, researchers have observed significant increases in the biosynthesis of valuable compounds^22^. The application of elicitors such as jasmonic acid and salicylic acid has been shown to modulate biosynthetic pathways, leading to higher yields of steviol glycosides, the primary sweetening agents in Stevia. Both acids are phytohormones that serve as signaling molecules in stress response and plant defense with subsequent enhancement of metabolite accumulation in response to pathogen attack or wounding^22^. Plant growth regulators are frequently used during plant propagation and are central to protocol success. For instance, cytokinins like BAP (6-benzylaminopurine) are often applied to induce shoot proliferation, while auxins such as 2,4-D (2,4-dichlorophenoxyacetic acid) and NAA (naphthaleneacetic acid) are used for root formation or callus induction based on the propagation pathway. Culture conditions, such as temperature, photoperiod, and subculture intervals, have also been reported to enhance metabolite profiles and shoot multiplication rates^23,24^. Additionally, plant tissue culture methods have been utilized to enhance the production rate of secondary compounds without the limitations imposed by external environmental factors^22^. These findings underscore the potential of biotechnological approaches to improve both the agronomic traits of plants and the production of steviol glycosides. Several previous studies have reported the production of steviol glycosides from *S. rebaudiana* using both direct and indirect micropropagation methods^25–27^. However, none have focused on time as a variable for accelerating steviol glycoside accumulation or incorporated quantitative comparison of metabolite production between different regeneration strategies. Therefore, this study was designed to establish a time-efficient protocol for callus induction and regeneration of *S. rebaudiana* via both direct and indirect micropropagation by optimizing certain variables specifically, the type and concentration of plant growth regulators, the type of explants used, and the culture conditions. Subsequently, both qualitative and quantitative assessments of the differences in metabolite profiles between the propagation-derived lines from a single Egyptian genotype and the soil-grown plant was performed using UPLC-MS coupled with multivariate data analysis. These investigations were conducted to achieve the ultimate objective of defining the optimum approach that yields the highest steviol glycoside content within the shortest cultivation period.

## Results and discussion

### In vitro indirect regeneration and callus formation of *S. rebaudiana*

**Fig. 1:**
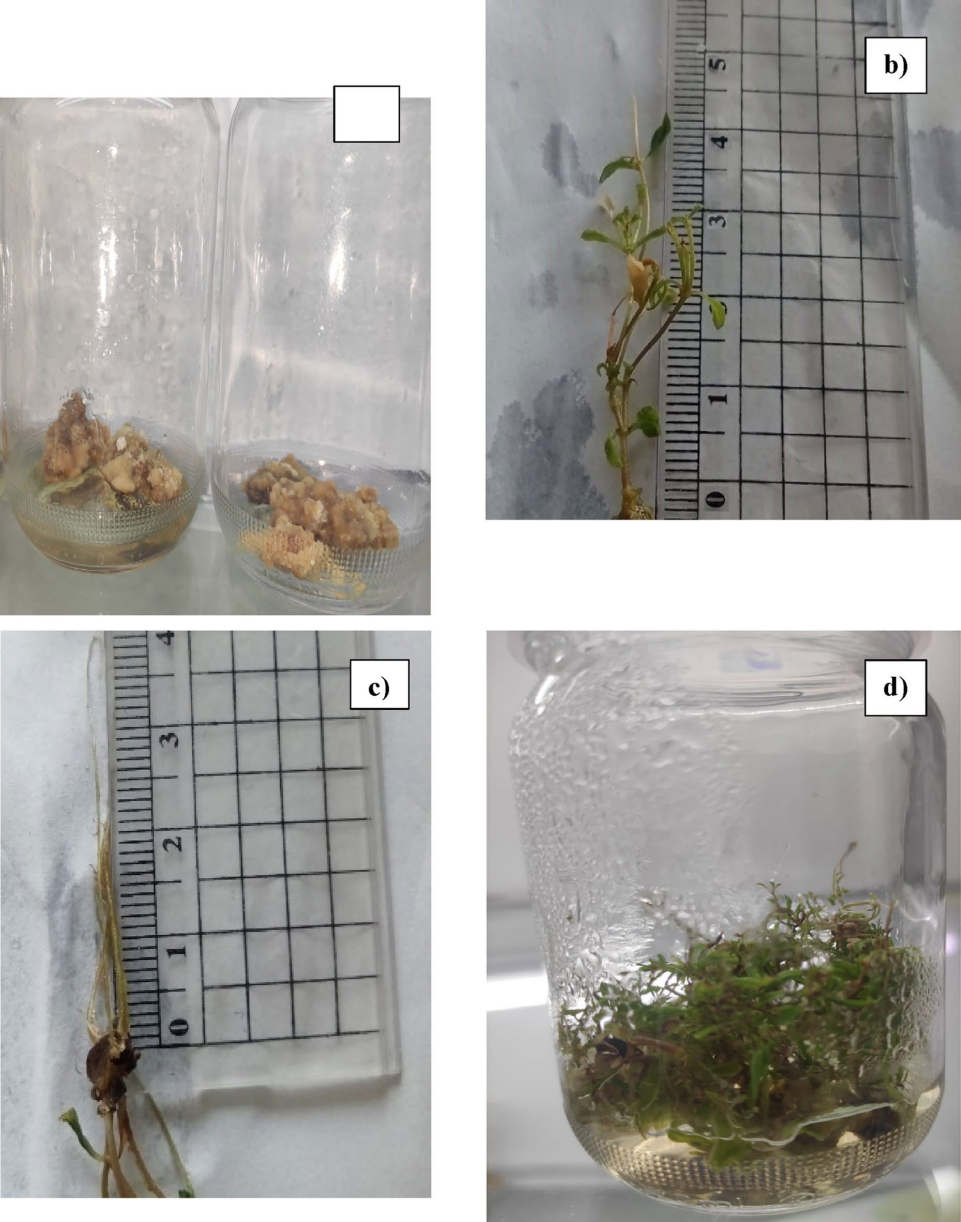
The indirect micropropagation of *S. rebaudiana*, (**a**) Induced Callus was regenerated in MS media supplemented with 1 mg/L of BAP and 2 mg/L Kin. (**b**) In vitro shoot of *S. rebaudiana* was regenerated on MS media (3.3 mg/L) fortified by 2 mg/L BAP, and 0.1 mg/L thiamine HCl, and 10 mg/L myo-inositol via indirect micropropagation. (**c**) In vitro root of *S. rebaudiana* was regenerated on MS media (2.2 mg/L) fortified by 2 mg/L IBA, and 0.5 mg/L thiamine HCl, and 10 mg/L myo-inositol via indirect micropropagation. (**d**) The regenerated plant of *S. rebaudiana* via indirect micropropagation of 28 weeks old.

Callus induction is the initial phase of indirect micropropagation in the tissue culture technique^20^. PGRs, like auxins and cytokinins in varying concentrations, are crucial for initiating callus formation, promoting its growth, and enhancing production of secondary metabolites^20^. The sterilized explants of *S. rebaudiana* stem parts were placed onto an MS medium fortified with various concentration of PGRs as follows: Kin (1, 2, and 3 mg/L), and BAP (1 mg/L), and NAA (1 mg/L). After 4 weeks, they were subcultured in the same media with the same combinations. The optimal callus formation was accomplished after eight weeks of incubation using fortified combinations of 1 mg/L of BAP and 2 mg/L Kin, producing a high percentage of callus capacity with a maximum biomass of 46.5 mg as shown in Fig. 1. The obtained calli is friable and yellowish-brown in color. However, the lowest callus mass was 12.6 mg, fortified by 3 mg/L of n NAA with 70% callus capacity. All results are provided in Table 1.

**Table 1:** Impact of plant growth regulators on the frequency of callus growth and the increase of callus weight.

The combinations of plant growth regulators (PGR)			Callus capacity (%)	Callus mass (mg)	
BAP (mg L^− 1^)	Kin (mg L^− 1^)	NAA (mg L^− 1^)		After 4 weeks	After 8 weeks
1	1	0	95	12.10 ± 0.38^b^	22.5 ± 0.29^c^
1	2	0	100	19.67 ± 0.35^e^	46.5 ± 0.39^g^
1	3	0	90	11.43 ± 0.22^b^	21.3 ± 0.18^b^
1	0	1	100	15.43 ± 0.35^d^	25.5 ± 0.29^d^
1	0	2	100	19.70 ± 0.15^e^	31.6 ± 0.23^e^
1	0	3	90	15.20 ± 0.12 ^d^	33.4 ± 0.11^f^
0	0	1	70	12.07 ± 0.17^b^	21.6 ± 0.20^bc^
0	0	2	85	13.20 ± 0.12^c^	45.9 ± 0.64^g^
0	0	3	70	8.33 ± 0.24 ^a^	12.6 ± 0.58^a^

*The values in the tables are means ± standard error, followed by different lowercase letters in each column under the same treatment represent the statistically significant differences at *p* ≤ 0.05 level. BAP; benzyl adenine, Kin; Kinetin, NAA; 1-Naphthaleneacetic acid.

*Callus capacity: it is the percentage of the formed callus, calculated through the following formula: (explant with cells of callus/total number of explants) × 100%), as it is known as the percentage of callus formed.

### Shoot initiation via indirect micropropagation

After four weeks of subculturing leaf-derived callus on MS media enriched with diverse concentrations of PGR such as BAP, and vitamins as Myo-Inositol, Thiamine HCl, and Pyridoxine-HCl. The results indicated that MS media fortified with 1.5 mg/L BAP with 15 mg/L Myo-Inositol, 1 mg/L of Thiamine HCl and 0.1 mg/L of Pyridoxine-HCl was the most effective. This combination resulted in the longest shoot length (1.53 ± 0.09 cm) and the highest number of shoots (19.8 ± 0.03). Conversely, the MS media enriched with BAP (0.5 mg/L), Myo-Inositol (5 mg/L), produced the shortest shoot length (0.26 ± 0.05 cm) and the lowest number of shoots (7.6 ± 0.02). A detailed overview of the results is presented in Table 2.

**Table 2:** In vitro culture medium used for shoot initiation of *S. rebaudiana* via indirect micropropagation using stem as explants after 4 weeks.

PGR	Vitamins			Mean number of shoots (nodes) per explants	Mean shoot length (cm)
BAP (mg/L)	Myo-inositol (mg/L)	Thiamine HCl (mg/L)	Pyridoxine-HCl (mg/L)		
0.5	5	0	0	7.6 ± 0.02^a^	0.26 ± 0.05^a^
0.5	5	0.5	0.1	12.2 ± 0.02^b^	0.46 ± 0.05^b^
1	10	0.5	0.1	13.8 ± 0.03^c^	0.66 ± 0.03^c^
1	10	1	0.1	15.2 ± 0.02^d^	1.13 ± 0.02^d^
1.5	15	1	0.1	19.8 ± 0.03^e^	1.53 ± 0.09^e^

*The values in the tables are means ± standard error, followed by different lowercase letters in each column under the same treatment represent the statistically significant differences at *p* ≤ 0.05 level. BAP, benzyl adenine.

### Shoot multiplication of *S. rebaudiana* via indirect micropropagation

The shoot multiplication stage was achieved, after 13 weeks, by supplementing MS media with varying concentrations of BAP, Myo-inositol, Thiamine HCl (mg/L), and Pyridoxine HCl, that subcultured occurring every 4 weeks. The outcome indicated that the MS medium enriched with BAP at 2 mg/L, and Myo-Inositol at 10 mg/L, Thiamine HCl at 1 mg/L and Pyridoxine-HCl at mg/L 0.1 was most effective, showing the longest shoot length (2.90 ± 0.03 cm), and the highest number of shoots (26.20 ± 0.86) as shown in Fig. 1. Conversely, the media fortified with BAP at 1 mg/L, Myo-Inositol at 5 mg/L, resulted in the shortest shoot length (0.89 ± 0.02 cm) and the lowest number of shoots (8.60 ± 0.51). All the data are presented in Table 3.

**Table 3:** In vitro culture medium used for shoot multiplication of *S. rebaudiana* through indirect micropropagation for 13 weeks.

PGR	Vitamins			Number of shoots per explants	Mean shoot length (cm)
BAP (mg/L)	Myo-Inositol (mg/L)	Thiamine-HCl (mg/L)	Pyridoxine-HCl (mg/L)		
1	5	0	0	8.60 ± 0.51^a^	0.89 ± 0.02^a^
1	5	0.5	0	10.80 ± 0.58^b^	0.095 ± 0.01^b^
1	5	0.5	0.1	17.20 ± 0.73^c^	1.04 ± 0.03^c^
2	10	0.5	0.1	22.60 ± 0.68^d^	1.57 ± 0.02^d^
2	10	1	0.1	26.20 ± 0.86^e^	2.90 ± 0.03^e^

*The values in the tables are means ± standard error, followed by different lowercase letters in each column under the same treatment represent the statistically significant differences at *p* ≤ 0.05 level. BAP, benzyl adenine.

### Root multiplication of *S. rebaudiana* via indirect micropropagation

To achieve the rooting, well-grown shoots measuring 1–1.5 cm were transmitted to half-strength MS medium (2.2 g) fortified with various concentrations of IBA (0.5, 1, and 2 mg/L), thiamine-HCl (0.5 and 1 mg/L), myo-Inositol (5 and 10 mg/L), and pyridoxine-HCl (0.1 mg/L), and cultured for three weeks. The most considerable result among the tested media was obtained by adding 1 mg/L Thiamine-HCl, 10 mg/L myo-inositol and 2 mg/L IBA to MS medium as shown in Fig. 1. However, the lowest rooting was achieved by MS media fortified with and IBA at 0.5 mg/L and myo-inositol at 5 mg/L. Table 4 summarizes all the findings.

**Table 4:** In vitro culture medium used for root multiplication of *S. rebaudiana* through indirect micropropagation for 3 weeks.

PGR	Vitamins			Mean root length (cm)
IBA (mg/L)	Myo-inositol (mg/L)	Thiamine HCl (mg/L)	Pyridoxine-HCl (mg/L)	
0	0	0	0	0.56 ± 0.02^a^
0.5	5	0	0	0.48 ± 0.02^b^
1	5	0.5	0.1	1.15 ± 0.03^c^
2	10	0.5	0.1	2.16 ± 0.02^d^
2	10	1	0.1	3.57 ± 0.04^e^

*The values in the tables are means ± standard error, followed by different lowercase letters in each column under the same treatment represent the statistically significant differences at *p* ≤ 0.05 level. IBA, indole-3-butyric acid.

### Shoot initiation of *S. rebaudiana* through direct micropropagation

The shoot initiation stage was highly achieved, after 28 days, by adding the explant to full-strength MS medium (4.4 g/L), enriched with 1.5 mg/ml BAP, and plant vitamins 0.5 mg/L Thiamine HCl, and 15 mg/L myo-Inositol. This combination showed the highest shoot number with (23.4) with the greatest root length with (1.9 cm). The lowest number of shoots (11.20) with the shortest length of shoots (0.5 cm) was achieved by MS medium fortified with 0.5 mg/mL BAP, and 5 mg/L myo-Inositol with the lowest length of shoots (1.9 cm). A summary of the results is provided in Table 5.

**Table 5:** In vitro culture medium used for shoot initiation of *S. rebaudiana* through direct micropropagation using axillary and apical meristem shoots for 4 weeks.

PGR	Vitamins			Mean Number of shoots (nodes) per explants	Mean shoot length (cm)
BAP (mg/L)	Myo-inositol (mg/L)	Thiamine HCl (mg/L)	Pyridoxine-HCl (mg/L)		
0.5	5	0	0	11.20 ± 0.49^a^	0.5 ± 0.10^a^
0.5	5	0.5	0.1	14.60 ± 0.69^b^	0.6 ± 0.19^a, b^
1	10	0.5	0.1	16.80 ± 1.32^b, c^	0.8 ± 0.16^b^
1	10	1	0.1	18.60 ± 1.08^c^	1.2 ± 0.25^c^
1.5	15	1	0.1	23.40 ± 0.93^d^	1.9 ± 0.26^d^

**The values in the tables are means ± standard error, followed by different lowercase letters in each column under the same treatment represent the statistically significant differences at *p* ≤ 0.05 level. BAP, benzyl adenine; IBA, indole-3-butyric acid.

### Shoot multiplication for *S. rebaudiana* via direct micropropagation

**Fig. 2:**
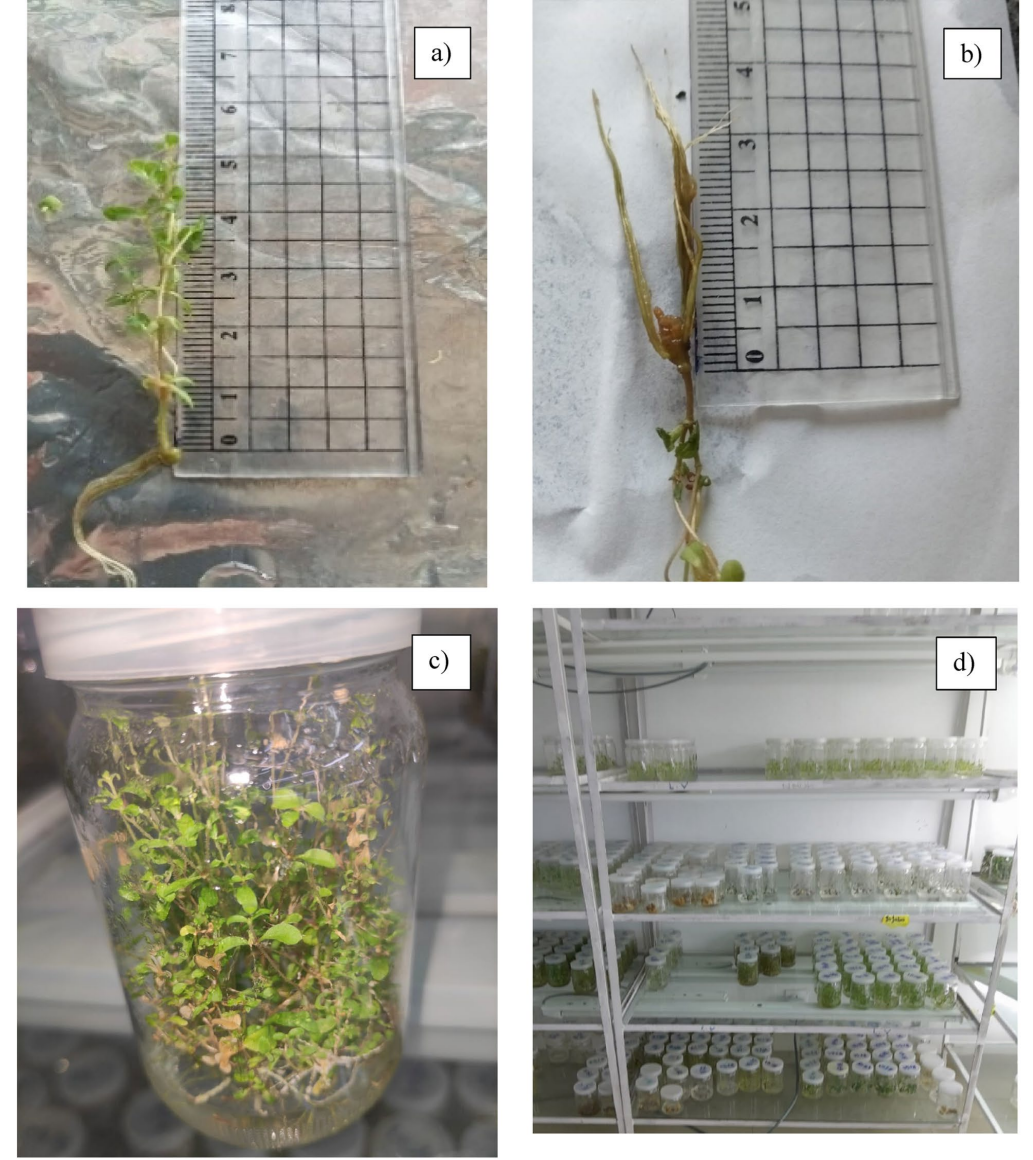
The direct micropropagation of *S. rebaudiana*, (**a**) In vitro shoot of *S. rebaudiana* regenerated on MS media (3.3 mg/L) fortified by 2 mg/L BAP, and 0.1 mg/L thiamine HCl, and 10 mg/L myo-inositol via direct micropropagation, (**b**) In vitro root of *S. rebaudiana* regenerated on MS media (2.2 mg/L) fortified by 2 mg/L IBA, and 0.5 mg/L thiamine HCl, and 10 mg/L myo-inositol via direct micropropagation, (**c**) The regenerated plant of *S. rebaudiana* via direct micropropagation of 20 weeks old, (**d**) The regenerated *S. rebaudiana* via direct and indirect micropropagation.

The lateral bud segments exhibited shoot multiplication after 13 weeks in different media containing varying concentrations of BAP, thiamine HCl, myo-Inositol, and pyridoxine-HCl (mg/L) with subculturing every 21 days. The number of Shoots formed per explant and the mean shoot length were recorded among all treatments. The optimal shooting was obtained by adding 2 mg/mL BAP, and 1 mg/L thiamine-HCl, and 10 mg/L myo-inositol in MS medium (3.3 g/L). This combination resulted in the highest shoot number per explant (32) and the greatest shoot length (1.73 cm) as shown in Fig. 2. In contrast, the lowest shoot multiplication was achieved by MS medium supplemented with 1 mg/mL BAP, and 5 mg/L myo-inositol, resulting in the lowest number of shoots per explant (11.60) and the shortest shoot length (1.10 cm). Table 6 provides a summery for all the results.

**Table 6:** In vitro culture media used for shoot multiplication of *S. rebaudiana* through direct micropropagation using axillary and apical meristem shoots for 13 weeks.

PGR	Vitamins			Number of shoots per explants	Mean shoots length (cm)
BAP (mg/L)	Myo-inositol (mg/L)	Thiamine- HCl (mg/L)	Pyridoxine-HCl (mg/L)		
1	5	0	0	11.60 ± 0.51^a^	2.11 ± 0.03^a^
1	5	0.5	0.1	21.80 ± 0.86^b^	3.27 ± 0.07^b^
2	10	0.5	0.1	29.60 ± 0.51^c^	4.18 ± 0.03^c^
2	10	1	0.1	32.0 ± 0.07^d^	5.72 ± 0.05^d^

*The values in the tables are means ± standard error, followed by different lowercase letters in each column under the same treatment represent the statistically significant differences at *p* ≤ 0.05 level (They are ascertained using the Post Hoc-Duncan test and one-way analysis of variance (ANOVA). BAP, benzyl adenine.

### Root multiplication of *S. rebaudiana* via direct micropropagation

Well-grown shoots, measuring 1.5–2 cm, were transferred to half-strength MS medium (2.2 g/L) fortified with different concentrations of IBA (0.5, 1, and 2 mg/L). The most significant result among the tested media was achieved by adding 0.1 mg/L of pyridoxine-HCl, 1 mg/L of thiamine-HCl, and 10 mg/L of myo-inositol and 2 mg/L of IBA, with subculturing for just 21 days, as shown in Fig. 2. On the other hand, the lowest rooting was achieved with the MS medium fortified with 5 mg/L of myo-inositol and 0.5 mg/L of IBA. The results are provided in Table 7.

**Table 7:** In vitro culture medium used for root multiplication of *S. rebaudiana* via axillary and apical meristem shoots after 3 weeks.

PGR	Vitamins			Mean root length (cm)
IBA (mg/L)	Myo-inositol (mg/L)	Thiamine HCl (mg/L)	Pyridoxine-HCl (mg/L)	
0.5	5	0	0	1.71 ± 0.2^a^
1	5	0.5	0.1	2.42 ± 0.03^b^
2	10	0.5	0.1	3.63 ± 0.02^c^
2	10	1	0.1	4.42 ± 0.03^d^

*Values are means ± standard error. Means within columns followed by different lowercase letters in each column under the same treatment represent statistically significant differences at *p* ≤ 0.05 level as determined by one-way analysis of variance (ANOVA) with Post Hoc-Duncan test. IBA, indole-3-butyric acid.

Our findings on shoot induction and callus regeneration align with previously reported protocols in *S. rebaudiana* and related medicinal species. For instance, the use of BAP and myo-inositol for shoot proliferation has also been shown to significantly enhance shoot numbers in *S. rebaudiana* under in vitro conditions^28^. The highest shoot induction in our study was achieved using 1.5–2.0 mg/L BAP, which is consistent with the cytokinin concentrations reported to be most effective in prior micropropagation studies.

We also observed variability in callus induction rates and shoot development across different types, which likely reflects intrinsic metabolic and hormonal differences. Rodriguéz-Páez et al. (2024) reported that variations in endogenous auxin and cytokinin levels among *S. rebaudiana* types influence morphogenic responses, a trend that was evident in our study as well, especially during indirect micropropagation^25^.

### UPLC-MS analysis of soil grown, callus, direct and indirect micropropagation of *Stevia rebaudiana*

**Fig. 3:**
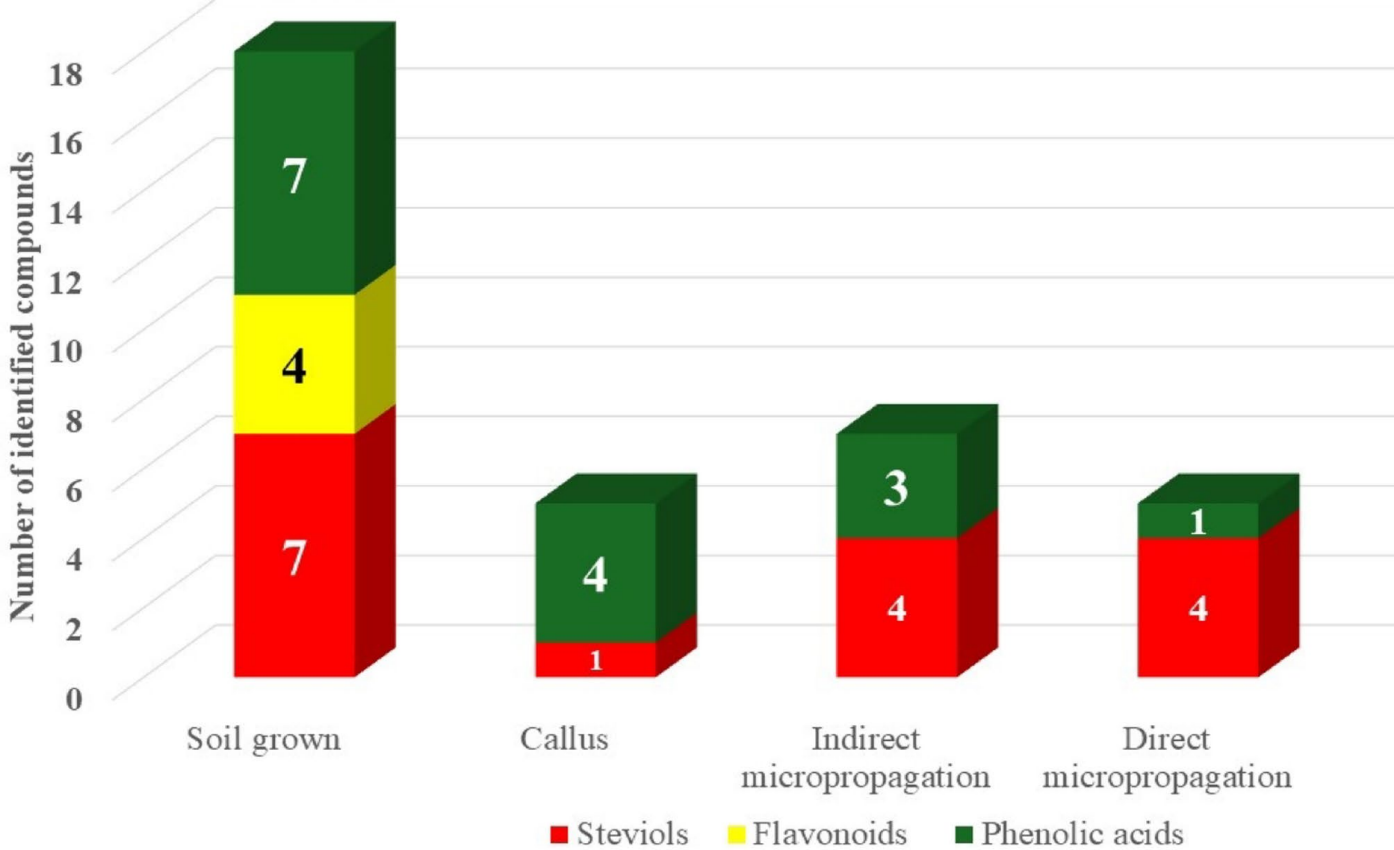
Number of tentatively identified compounds from *Stevia* (Soil-grown, callus, indirect and direct micropropagation).

The regeneration route—direct vs. indirect—impacted not only growth rates but also the accumulation of secondary metabolites, particularly steviol glycosides. Qualitative and quantitative UPLC-MS analysis revealed that directly regenerated plants exhibited total steviol glycoside profile more comparable to that derived from soil-grown plants, suggesting that direct regeneration preserves metabolic fidelity more effectively than callus-mediated regeneration. This finding supports the application of direct micropropagation for commercial-scale production of high-glycoside-content stevia (soil-grown, RDM, RINM, and callus with total area 35.95%, 23.87%, 8.58% and 3.18% respectively), offering both time efficiency and metabolic consistency. About 18 compounds were identified, comprising phenolics (seven phenolic acids, and four flavonoids), and seven terpenoids in all tested types of stevia. The number of tentatively identified compounds in all tested types of stevia are shown in Fig. 3.

### Qualitative profiling

#### Phenolic acids

Phenolic acids are considered the second most prevalent subclass of phenolic compounds, which are widespread in various parts of plants and regarded as a vital human dietary component^29^. Several pharmacological studies have highlighted the potential of phenolic acids as free radical scavengers and their subsequent beneficial impacts on human health^29^such as protection against the development of diseases like cancer, as well as antimicrobial, antiviral, antiallergic, antithrombotic, and anti-inflammatory properties^29^. Caffeoylquinic acids are a phenolic acid example with high scavenging activity significant antioxidant, considered strong metal chelators, and displayed strong inhibitory effects on lipid oxidation^30^. The UPLC-MS/MS analysis led to identification of six phenolic acids. In the four types of studied stevia Peak (6) with molecular ion peak at *m/z* 515 ^31^, was detected. The base peak at *m/z* 353 corresponds to the molecular ion peak, representing the loss of a caffeoyl moiety [M-162] ^−^. The fragment ion at *m/z* 191 corresponding to the base peak, represents the loss of another caffeoyl by breakage of the bond between quinic acid and caffeoyl moiety [M-162]^−^. In addition, it showed the presence of distinctive fragments ions at *m/z* 173 and 155, representing the consecutive loss of water moiety [M-18]^− 32^. This pattern of fragmentation is characteristic for 3,5-isomer compare to other di-caffeoylquinic acids^32^. It could be tentatively identified as 3,5-di-O-caffeoylquinic acid compared to the literature^33^. Several pharmacological studies report the beneficial effect of the compound. It exhibits several biological activities, including being a significant neuroprotective agent^34^antioxidant, anti-prostate cancer^35^ and wound healing activity^36^. Moreover, its antioxidant activity is generally stable in various cooking methods, making it a better option for usage as a natural antioxidant^37^. Peak (1) is present in all studied stevia, except for the direct micropropagated stevia, which gave molecular ion peak at *m/z* 353 ^31−^. The base peak at *m/z* 191 indicates loss of caffeoyl moiety [M-162]^−^ by cleavage of the bond between quinic acid and caffeoyl moiety. A common fragment ion at *m/z* 179 with higher intensity, corresponds to the loss of quinic moiety. based on the literature, peak (1) was annotated as 3-O-caffeoylquinic acid^32^.Previously, reported with a potent antioxidant^38^ and management of collagen-induced arthritis^39^. Peak (8) at *m/z* 515 ^31−^, has been found in all studied stevia except the regenerated with direct micropropagated. Fragment ion at *m/z* 353 corresponds to the loss of caffeoyl moiety [M-162]^−^. Other fragments at *m/z* 179 and 135 correspond to the detachment of the quinic acid moiety [M-162]^−^ and further loss of the moiety (CO2) from caffeoyl fragment. It displayed a base peak at *m/z* 173, that is characteristic for 4-acylated isomers of caffeoyl quinic acids. Previous results suggested that, peak (8) is annotated as 4, 5- di-*O*-caffeoylquinic acid^40^. Peak (5) at *m/z* 515 ^31−^, presented only on soil-grown and callus, exhibited a base peak at *m/z* 173 which is typical of 4-acylated isomers. Another characteristic fragmentation, at *m/z* 353 represent deprivation of caffeoyl moiety [M-162]^−^ and *m/z* 335 with high intensity represents the subsequent loss of water moiety [M-18]^−^. With comparing to the literature, it could be tentatively annotated as 3,4-di-*O*-caffeoylquinic acid^36^. It was reported with a various biological activity as, anti-prostate cancer activity^35^α-glucosidase inhibitory activity^41^anti-adipogenic^42^anti-inflammatory, antibacterial, antiviral, hypotensive, anticancer, hepatoprotective and potent antioxidant activity^43^. Peak (2) with molecular ion peak at *m/z* 353 ^31^, was presented only at soil-grown stevia. It exhibited a base peak at *m/z* 191 indicates the quinic acid residue. Another fragment at *m/z* 179 with lower intensity indicate elimination of quinic acid group, and subsequent fragments at *m/z* 161 indicate loss of water moiety [M-18]^−^. It could be deduced that peak (2) is defined as 5-*O*-caffeoylquinic acid compared to the literature^33^. Previous studied have shown its strong anti-microbial activity^44^anti-cancer^45^and improving lipid metabolism disorders^46^. Peak (15) and (18) with molecular ion peaks at *m/z* 359, and 367 ^31−^ present only on soil-grown stevia. Peak (15) showed a base peak at *m/z* 197, indicating the detachment of a hexosyl moiety [M-162]^−^ and other fragments at *m/z* 182, 167, and 153 consist with the fragmentation pattern of syringic acid. With Comparing to the literature, it was recognized as Syringic acid-O-hexoside^47^. Peak (18) with base peak at *m/z* 191 represents removal of quinic acid moiety [M-162]^−^ and other fragments at *m/z* 173 represents removal of water fragment [M-18]^− 48^. Based on previous data it was assigned as 5- O-feruloylquinic acid^49^. All results are summarized in Table 8.

### Flavonoids

**Fig. 4:**
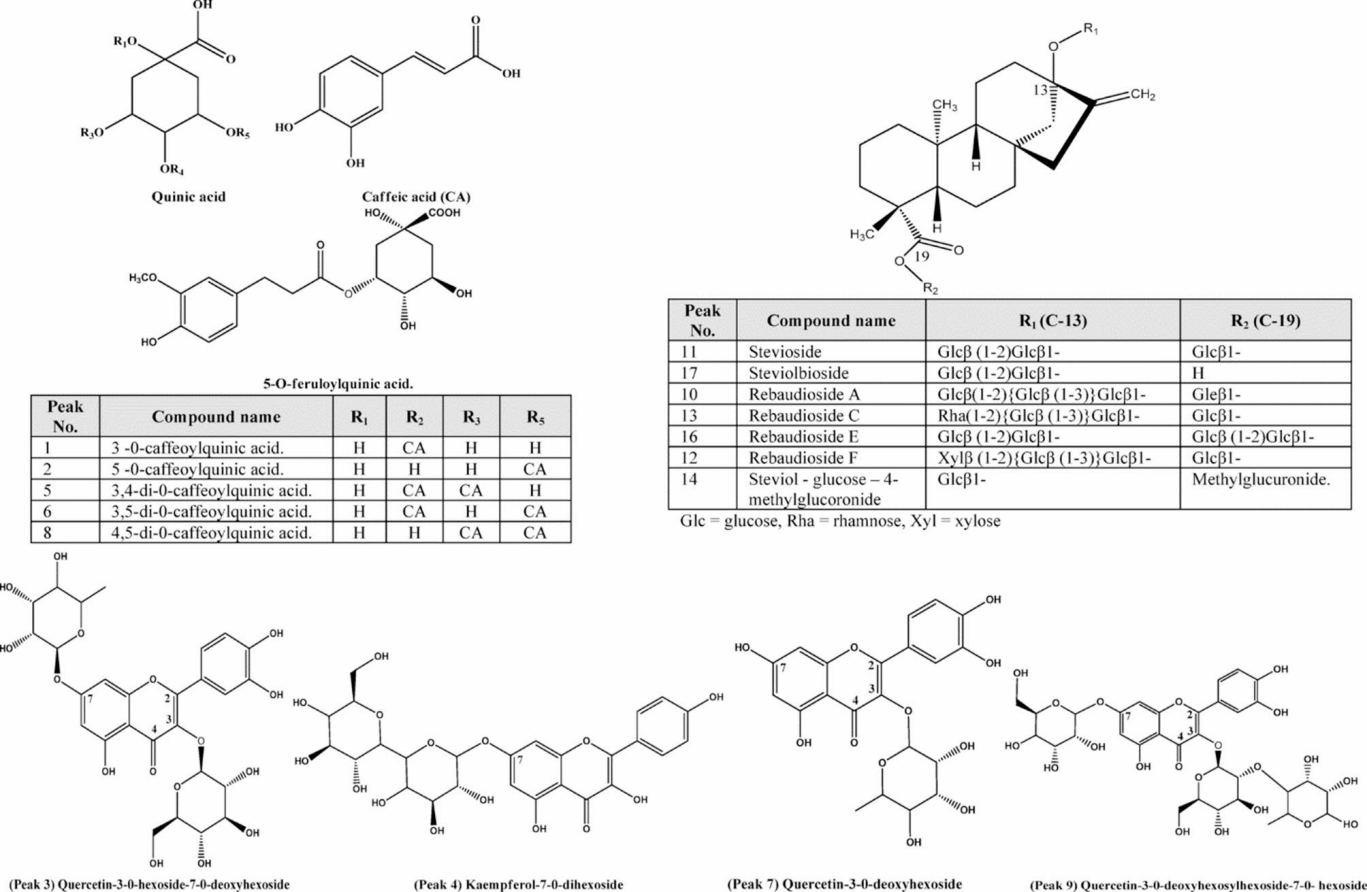
Chemical structures of tentatively identified phenolic compounds and steviol glycosides from *S. rebaudiana*.

Flavonoids are the most abundant natural polyphenolic compounds, which present in different parts of the plants organs, with a broad range of reported biological activities^50^. Their consumption is related with a variety of health-promoting benefits to the human being^50^. In this work all the identified flavonoids using UPLC-MS analysis method, were detected only in soil grown stevia. Peaks (3), (7), and (9) with molecular ion peak at *m/z* 609, 447, and 771 respectively, showed a distinctive fragment of quercetin aglycone at *m/z* 300, corresponding to quercetin aglycone skeleton. Other fragments at *m/z* 271 correspond to the detachment of (–CH_2_O), *m/z* 255 is related to the loss of a (–CH_2_O_2_) fragment, and *m/z* 179 produced by Retro Diels-Alder Fragmentation (RDA) breakage between (O-C_2_) and (C_2_-C_3_), enabling the deprivation of a (–C_7_H_6_O_2_) fragment (Fig. 4). The last distinctive fragment was at *m/z* 151, which was also a result of RDA cleavage, but this time, between (O-C_2_) and (C_3_–C_4_), followed by the loss of (–C_8_H_6_O_3_) (Fig. 4). Peak (3) with molecular ion peak at *m/z* 462 ^31^ showed that the deoxyhexose fragment was located at the 7-*O* position as the detachment of a glycosyl unit at the 7-*O* position is more preferred than the hexose unit was located at the (3-O) position. From previous results, comparing to literature it was tentatively identified as Quercetin-3-*O*-hexoside-7-*O*-deoxyhexoside compared to literature^51^. Peaks (7) showed the loss of deoxyhexose [M-146]^−^, which when compared to literature it was recognized as Quercetin-3-*O*-deoxyhexoside^52^which previously reported with antiviral activity^53^. Peak (9) with a fragment at *m/z* 609 [M-162]^−^, demonstrated the connection of a hexose unit at (7-O). An additional loss of fragment at *m/z* 301 [M-308]^−^ confirmed the exitance of a deoxy-hexosylhexose unit in the compound. Based on the previous data, it was assigned as Quercetin-3-*O*-deoxyhexosylhexoside-7-*O*-hexoside^52^. Peak (4) with molecular ion peak at *m/z* 609 ^31^, showed a distinctive fragment at *m/z* 447, that resembles the loss of hexose fragment. Meanwhile it showed a loss of [M-162] ^–^ owing to the hexose moiety, generating the base peak at *m/z* 285 corresponds to Kaempferol aglycone part. Based on these results it can be deduced that compound (4) assigned as Kaempferol-7-*O*-dihexoside as compared to literature^54^. All results are summarized in Table 8.

### Terpenoids

Terpenoids are a large class of naturally occurring secondary metabolites, with over 50,000 terpenoids identified, exhibiting diverse pharmacological actions and various medicinal applications^55^. More than 30 types of steviol diterpenoid glycosides were previously reported from *S. rebudiana*, which is characteristic of its sweetness compared to natural sweeteners^55^. The United States Food and Drug Administration (US-FDA) and the European Food Safety Authority (EFSA) have considered stevia leaf extract, with a daily dose 4 mg/kg of body weight of steviol glycosides equivalents, to be a safe herbal product^8^. This study employed UPLC-MS analysis, that led to preliminary identification of seven diterpene glycosides in the four stevia samples. Peak (11) with molecular ion peak at *m/z* 803 ^31−^ was detected in four types of the studied stevia with the diagnostic detachment of a glucose unit appearing at *m/z* 641, as the cleavage of the weaker ester linkage between the glucose unit and the carboxyl group at (C_−_19) (Fig. 4). Other fragments at *m/z* 479, and 317 indicate detachment of 2 glucose moieties. Based on these results it could be deduced that with comparing to literature identified as stevioside^56^. The most important steviol glycoside is stevioside which is non-toxic, thermally stable, low-calorie natural, and is mainly responsible for the sweetness of stevia with ca. 200–300 fold sweetener than sucrose^57^. Stevioside was reported with other physiological activities as being antihypertensive with intakes of 500 mg/day^58^antifibrotic^59^, and exhibited moderate tuberculostatic activity^60^immune regulation^61^antioxidant, anti-inflammatory, antidiabetic, hepatoprotective, antimicrobial, and anticancer^58^. Peak (10) and (13) were detected in all types of stevia studied except in callus. The molecular ion peak of (peak 10) at *m/z* 965 ^31−^, with distinctive fragmentation patterns at *m/z* 803, resulting from the removal of a hexosyl group. Further losses of the hexosyl units gave fragment ions at *m/z* 641, 479, and 317. It could be deduced that peak (10) was annotated as rebaudioside A compared to the literature^62^. Rebaudioside A is one of the most important steviol glycoside responsible for the sweetness of stevia with ca. 200–300 times more sweetener than sucrose^63^. It showed several biological activities as being antihyperglycemic^44^hepatoprotective effect^64^antioxidant^65^anti-caries^66^ and anti-obesity^58^. The presence of both stevioside and rebaudioside A in the plant is responsible for the aftertaste and ensures its efficacy as a sweetening agent^11^. The molecular ion peak of peak (13) at *m/z* 949 ^31−^ with fragmentation patterns *m/z* 787, and 625, resulting from the consecutive loss of a dihexosyl moiety. Another fragmentation pattern at *m/z* 479, resembles the removal of rhamnose moiety. With comparing these results to the literature it was annotated as rebaudioside C^67^. Peak (14) with at *m/z* 935 ^31−^, presents in both soil-grown and direct micropropagation stevia, exhibiting fragment at *m/z* 687 and base peak at *m/z* 479, indictive of the detachment of 4-*O*-methylglucuronide. It implies that the 4-*O*-methylglucuronide was attached to (C_−_19) of the steviol aglycone, through the methyl group, not hydroxyl one, afterward the peak at *m/z* 317 that indicates the removal of hexose unit at the (C-13) position. Based on this information, compared to literature data, it was annotated as Steviol glycoside containing hexose and 4-*O*-methylglucuronide units^54^. Peaks (12), (16), and (17) were detected only in soil-grown stevia. Peak (12) at *m/z* 935 ^31−^, with fragmentation patterns at *m/z* 773, in accordance with the detachment of a hexosyl unit [M-162]^−^ from (C-13), showed the loss of up to two hexosyl fragments resulting in peak ions at *m/z* 611, and 479 from (C-19) (Fig. 4). Results with comparing to literature data annotated as rebaudioside F^54^. Peak (16) at *m/z* 965 ^31−^ with a base peak at *m/z* 641 aligned with the loss of a dihexosyl moiety [M-324]^−^ from (C-19) linked to the carboxylic group. Additional fragment ions at *m/z* 479 and 317 are resulted from sequential losses of hexosyl units from (C-13) (Fig. 4). With comparing to literature data it was annotated as rebaudioside E^54^. Peak (17) displayed a molecular ion peak at *m/z* 641 ^31 −^, with distinctive fragments 479, and 317 corresponds to the successive losses of hexosyl moieties from (C-13). When compared to literature data, it was identified as Steviolbioside^68^which was previously reported to have moderate tuberculostatic activity^60^. All results are summarized in Table 8.

### Quantitative profiling

Quantitative profiling of *S. rebaudiana* propagated via callus-induction, direct, as well as indirect regeneration and soil-grown protocols was conducted using UPLC-MS/MS to complement the qualitative identification of steviol glycoside and phenolic constituents. The analysis was performed by comparing their relative peak areas of the total ion chromatogram in each sample, as summarized in Table 8.

### Steviol glycosides

Among the primary sweet steviol glycosides identified in *S. rebaudiana*, stevioside at peak 11, showed the highest abundance in all samples. Its greatest relative peak area was in RDM, followed by soil-grown, RINM, and callus-derived samples (13.17, 11.81, 6.69, and 3.18%, respectively). Similarly, rebaudioside A, peak 10 revealed an area of 5.71% in RDM, which was remarkably greater than that in RINM (1.28%), meanwhile it was unobserved in the callus. Previous studies reported a range of stevioside content from 5 to 15% of dry leaf weight while that of rebaudioside was between 2 and 6% in a soil-grown plant^69^. Rebaudioside C presented highly in soil-grown compared to RDM, and RINM with total area 13.82, 4.36, and 0.61 respectively, meanwhile it was absent in the callus. Additionally, rebaudioside C and steviolbioside, peaks 13 and 17 were significantly denoted in soil-grown and RDM samples, with rebaudioside C accounting for 13.82 and 4.36% respectively, while it was absent in callus and RINM samples. These yields approach specifically, that of RDM samples, highlighting the close alignment in sweeteners profiles between the direct regeneration strategy and the conventional cultivation method. Thus, it may be considered as a viable propagation technique for natural sweetener production.

### Phenolic compounds

Beyond sweeteners, *S. rebaudiana* was rich in several key phenolic acids and flavonods contributing to its medicinal prospectives. UPLC-MS/MS analysis showed substantial differences in the accumulation of those compounds between various propagation techniques. Remarkably, 4,5-di-*O*-caffeoylquinic acid, peak 8 was the most abundant in callus (29.62%) and soil-grown plants with peak areas of (29.62 and 15.54%), respectively while it was significantly minor or absent in RINM and RDM samples. In contrast, 3,5-di-*O*-caffeoylquinic acid, peak 6 was predominant in RINM and callus (28.57 and 13.48%, respectively), but comparatively low in soil-grown and RDM samples (2.86 and 2.03%, respectively). Notably, 3-*O*-caffeoylquinic acid, peak 1 was observed in all samples except for RDM, with the greatest quantity in callus accounting for (26.04%). These results were consistent with prior studies, which documented high phenolic and flavonoid content in soil-grown stevia and reduced accumulation in RDM^70^suggesting the promising efficacy of direct micropropagation technique to prioritizes the biosynthesis of steviol glycosides, possibly at the expense of that some phenolic constituents.

### Metabolic implications for sweetener production

Qualitative and quantitative phytochemical analysis of four *S. rebaudiana* samples, including callus-derived, directly, as well as indirectly regenerated, soil-grown plants, revealed that metabolite content varied significantly in response to different propagation techniques. Previous studies have described that growth cycles, transplantation stress, and agroclimatic environments such soil composition may give rise to marked alterations in the accumulation of secondary metabolites, such as steviol glycosides, flavonoids, and phenolic acids^15,71^. For example, the content of steviol glycoside tends to decrease directly after transplantation as a result of metabolic reallocation toward root establishment and stress recovery however increase once stevia stabilizes and re-initiate the active state of growth. Consistently, the flavonoid and phenolic biosynthesis can be moderated by stresses either biotic or abiotic, including salinity, drought, and by cultivation practices such as conventional vs. organic farming^16^. These fluctuations in metabolic pathways must be taken into account during phytochemical standardization of stevia-derived products and are important consideration upon selecting the proper propagation method whether for medicinal value or sweetener outcome.

Despite the fact that *S. rebaudiana* thrives in relatively high humidity and moderate temperatures of subtropical climates, Egypt’s arid to semi-arid environments, characterized by low relative humidity and high evapotranspiration rates, face agronomic challenges with respect to field cultivation. Remarkably, stevia is sensitive to water stress, mainly during sprouting and early developmental stages, leading to reduced biomass and steviol glycoside production. However, its overall water demand is markedly lesser than that of beet or sugarcane, introducing stevia as a potential sustainable alternative under Egypt’s growing water insufficiency. Upcoming studies should prioritize strategies customized to Egypt’s agroclimatic constraints including selecting drought-tolerant genotypes, implementing advanced domestication plans, adjusting effective irrigation structures, and applying controlled-environment planting. In vitro culture, and hydroponic farming could also mitigate extreme humidity and temperature fluctuations. The enhanced regeneration protocol established by this study, with its improved metabolite production rates, was planned to support from small to large-scale stevia agriculture. This strategy had the ability to generate the required transplants for 1–2 hectares/cycle of stevia. Moreover, centralized tissue culture facilities can provide scaling up, address national needs for high-quality material, and support Egypt’s policy to condense sugar imports.

**Table 8:** Tentatively identified secondary metabolites in *Stevia rebaudiana* soil grown (S), callus (C), regenerated with direct micropropagation plant (RDM), and regenerated with indirect micropropagation (RINM) using UPLC/MS analysis.

Peak no.		*R*_t_ (min)	Molecular weight	^31−^	m/z fragments	Tentatively identified compound	Stevia rebaudiana total area %			
							S	C	RINM	RDM
Phenolics										
Phenolic acids										
1	1.86		354	353	353, 191, 179, 155, 135	3 -0-caffeoylquinic acid.	1.62	26.04	4.84	--
2	1.98		354	353	353, 191, 179, 173, 161, 135	5 -0-caffeoylquinic acid.	5.22	--	--	--
5	6.32		516	515	353, 334, 191, 179, 173, 161, 155, 135	3,4-di-0-caffeoylquinic acid.	3.2	15.28	--	--
6	6.39		516	515	353, 191, 179, 173, 155	3,5-di-0-caffeoylquinic acid.	2.86	13.48	28.57	2.03
8	6.68		516	515	352, 191, 179, 173, 154, 135	4,5-di-0-caffeoylquinic acid.	15.54	29.62	8.07	--
15	9.29		360	359	197, 182, 167, 153, 38,	Syringic acid-0-hexoside.	2.49	--	--	--
18	14.41		368	367	191, 173	5-0-feruloylquinic acid.	3.02	--	--	--
Flavonoids										
3	5.61		610	609	462, 447, 300, 301, 299, 271, 255, 179, 151	Quercetin-3-0-hexoside-7-0-deoxyhexoside	1.34	--	--	--
4	5.70		610	609	447, 285.	Kaempferol-7-0-dihexoside	2.4	--	--	--
7	6.44		448	447	301, 300, 271, 255, 179, 151,	Quercetin-3-0-deoxyhexoside	2.91	--	--	--
9	6.81		772	771	609, 301, 300, 299, 271, 179, 151	Quercetin-3-0-deoxyhexosylhexoside-7-0- hexoside	1.03	--	--	--
Terpenoids										
10	7.19		967	965	803, 641, 479, 413, 317	Rebaudioside A	3.08	--	1.28	5.71
11	8.39		804	803	641, 640.	Stevioside	11.81	3.18	6.69	13.17
12	8.62		937	935	773, 611, 479, 413	Rebaudioside F	3.2	--	--	--
13	8.76		951	949	787, 625, 479, 413, 317	Rebaudioside C	13.82	--	0.61	4.36
14	9.19		688	687	641, 479, 317.	Steviol + glucose + 4 methylglucoronide	1.22	--	--	0.63
16	9.63		966	965	641, 479.62, 317.11	Rebaudioside E	0.92	--	--	--
17	9.74		642	641	641, 479, 317	Steviolbioside	1.9	--	--	--

### Multivariate data analysis of secondary metabolites identified via UPLC-MS in *Stevia rebaudiana* soil grown (s), callus (C), regenerated by direct micropropagation plant (RDM), indirect micropropagation (RIDM)

**Fig. 5:**
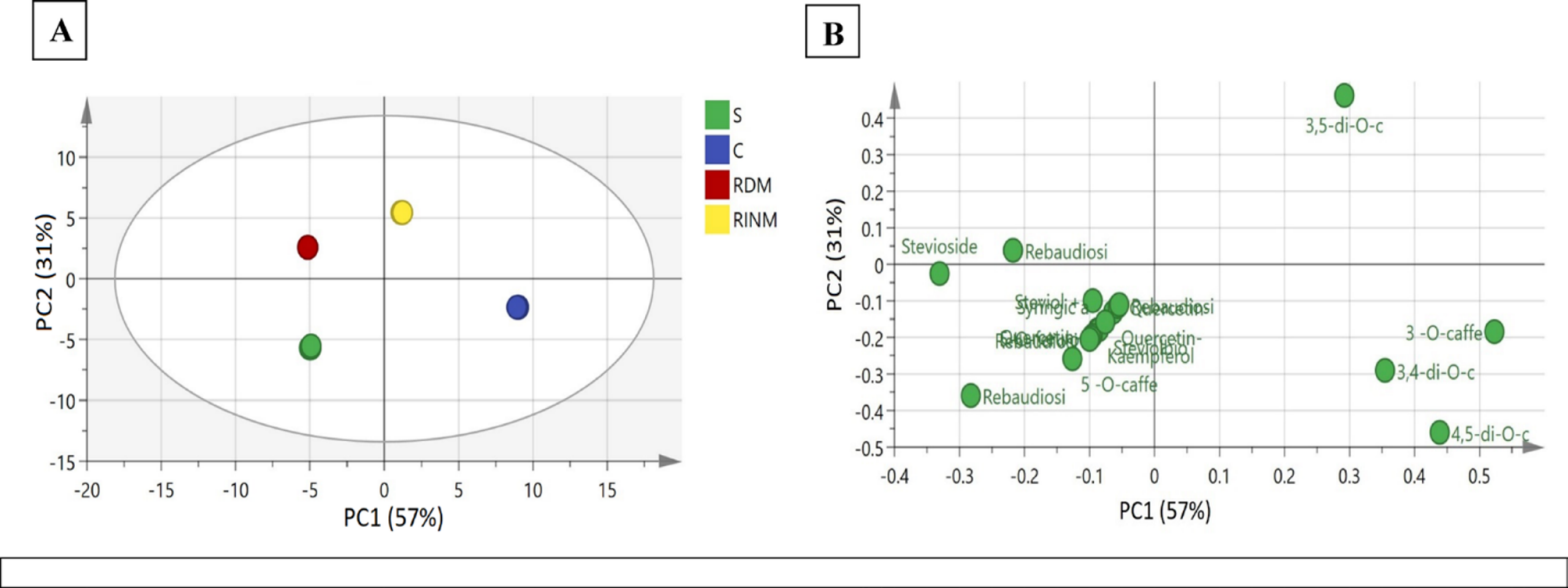
Principal component analysis (PCA) of secondary metabolites in *Stevia rebaudiana* soil grown (s), callus (C), regenerated *via* direct micropropagation plant (RDM), and regenerated indirect micropropagation using UPLC/MS-MS analysis. (**a**) PCA score plot of PC1 vs. PC2 scores, (**b**) The loading plot for PC1 and PC2. The metabolite clusters are positioned in a two-dimensional space at distinct locations defined by two principal component PC1 = 57% and PC2 = 31%.

Multivariate analysis is an effective statistical used to analyze data sets containing more than one variable^72^. It enables the creation of classifications by offering information in form of factual facts^72^. Principal component analysis (PCA) is one of the most used exploratory tools in multivariate analysis. It is employed to identify similarities and uncover hidden patterns among samples, particularly when relationships within the data and sample grouping is still unclear. The primary use of PCA is to differentiate between UPLC-MS metabolite profiles of the four studied plant groups-derived from different propagation methods; RDM, RIDM, callus-derived and soil-grown. The recorded metabolites were mainly steviol glycosides, phenolic acids and flavonoids. PC1 accounts for 56.9%, while, PC2 accounts for 31.2%. The PC1/PC2 score plot showed that the four distinct clusters are formed and distributed across the four plot regions, corresponding to the four samples. An examination of the loading plot showed discrimination between callus and regenerated stevia by indirect micropropagation on the positive side versus the soil-grown and directly micropropagated stevia. The upper right side of the loading (Positive PC1 values) suggests that the callus of stevia is mainly enriched with 3,5 di-*O*-caffeoylquinic acid (Peak 6). Meanwhile, the lower right side of the loading (Positive PC1 values) for regenerated stevia by indirect micropropagation, is enriched by 3-O-caffeoylquinic acid (Peak 1), and 3, 4 di-O- caffeoylquinic (Peak 5) as shown in figure (5). On the contrary, on the plot’s left side (negative PC1 values), steviol glycosides are positioned between the upper and lower sides of rebaudioside A (Peak 10) and stevioside (Peak 11), and between soil-grown and regenerated stevia by direct micro propagated, both of which are responsible for the sweetness of the stevia. On the other hand, on the plot’s lower left side (negative PC1 values), other steviol glycosides, such as rebaudioside (F, C, and E) peaks (12, 13, 16), steviolbioside (Peak 17), and other phenolic compounds are abundant in soil-grown stevia as shown in Fig. 5. The current results from UPLC-MS coupled with PCA affirm that the direct micropropagation is closely related to soil-grown for being for use as a natural sweetener.

## Conclusion

During the last years, Egypt has experienced a sugar shortage problem due to an imbalance of the local supply driven largely by the ongoing water crisis. In response, efficient *S. rebaudiana* regeneration protocols including callus induction and direct as well as indirect micropropagation strategies were established. Among the tested conditions, MS medium supplemented with 2.0 mg/L BAP and 0.5 mg/L NAA accomplished the highest shoot induction frequency (91.6%), while 2.0 mg/L 2,4-D induced callus formation (86.3%). Rooting was most effective on half-strength MS medium containing 1.0 mg/L IBA, achieving a 94.4% rooting rate. UPLC-ESI-MS/MS analysis, coupled with PCA and PLS-DA, revealed significant variations in metabolite profiles among four lines of *S. rebaudiana*. Notably, direct micropropagated stevia showed the highest accumulation of rebaudioside A and stevioside, the commercially valuable sweeteners, compared to callus, indirect micropropagated and soil-grown. These results confirm that this technique favored the production of steviol glycoside and offered the optimal balance between metabolite yield and growth duration, presenting it for scalable stevia production of stevia. In the contrary, callus and indirect regeneration strategies seems to enhance or retain some phenolic intermediates. These differences in metabolite accumulation patterns may be attributed to the induced stress in early precursors under in vitro settings and underscore the impact of propagation strategy on modulating the biosynthesis of secondary metabolite. Overall, the optimized micropropagation protocol and related metabolomic insights offer valuable tools for enhancing the sustainable production of high-quality stevia lines suitable for the Egyptian agroclimatic conditions and resource-limited agriculture.

## Materials and methods

### Materials

#### Plant material

**Fig. 6:**
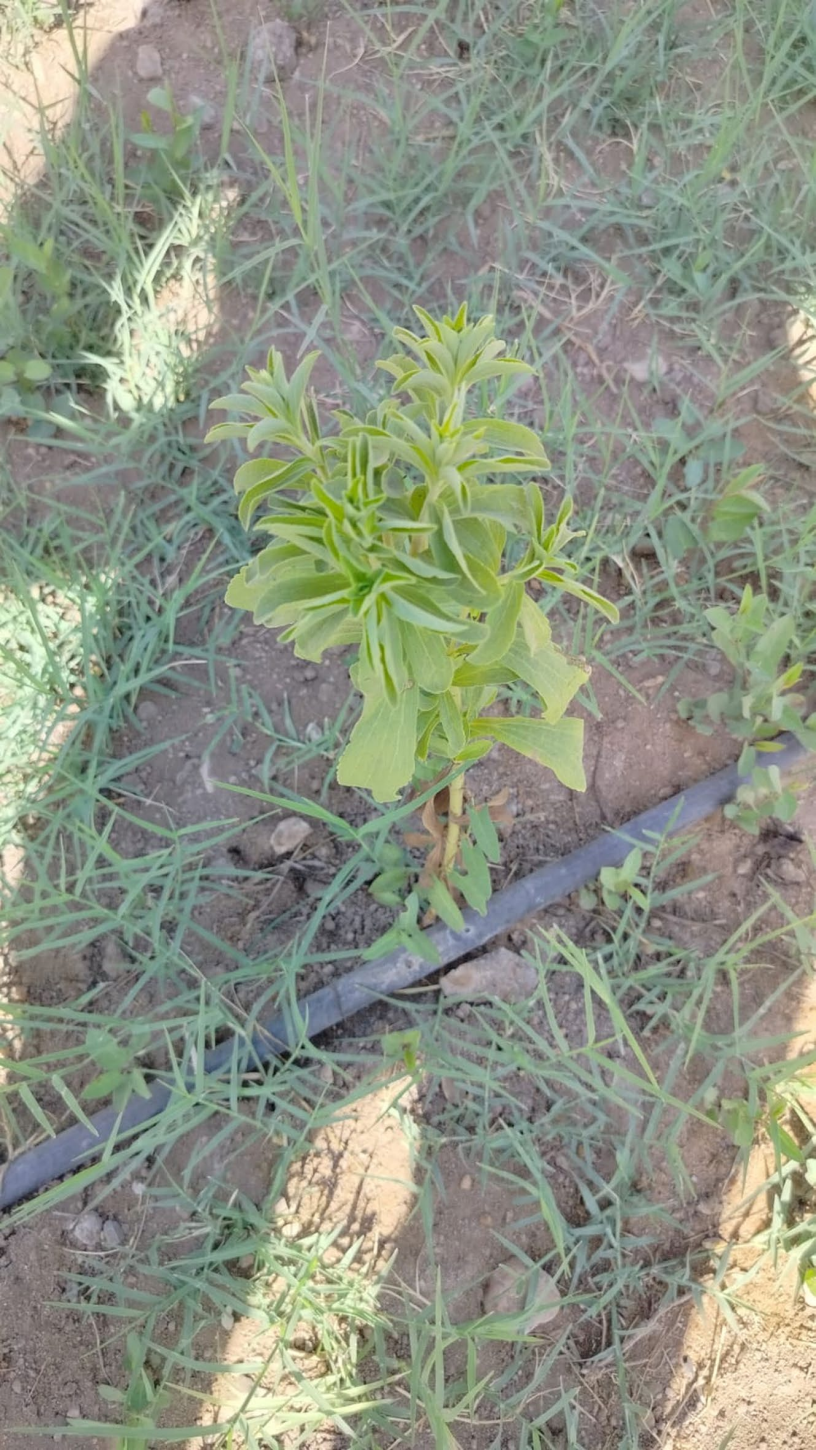
Soil-grown *Stevia rebaudiana*, 210 days old, from SEKEM Farm, Egypt.

A single cultivated genotype of *Stevia rebaudiana* was collected from SEKEM Farm in July 2023, Egypt (30° 24ʹ 58″ N 31°38ʹ 23″ E), at 180 days old, as shown in Fig. 6. Permission for the collection of *S. rebaudiana* was obtained from SEKEM Farm prior to sampling. The plant material was formally identified and authenticated by one of the authors; Dr. Fekria M. Ali, Faculty of Organic Agriculture, Heliopolis University. The voucher specimens were placed in the Herbarium of the Department of Pharmacognosy, Faculty of Pharmacy, Heliopolis University, in Egypt under name (HELIOSR 100145). 100 g of *S. rebaudiana* were dried in the shade for two days, then macerated in 500 ml of 100% methanol four times until exhaustion, using a sonicator for 30 min each. Then, it was filtered and evaporated using rotary evaporator at 35 °C, yielding a crude extract of 4.8 g. Plant tissue culture trails were conducted in the Plant Tissue Culture Lab, at the Faculty of Organic Agriculture, Heliopolis university, Egypt.

#### Materials for tissue culture

Commercially available Murashige and Skoog (MS) powdered medium (Duchefa, Germany), purified agar for plant tissue culture, Bioworld, USA. A commercial hypochlorite solution (Colorox^®^), which contains 5% sodium or calcium hypochlorite. Sucrose (Adwic, A. R. E.), and dilute HCl and KOH solutions for pH adjustment (Adwic, A. R. E.). The used plant growth regulators (PGR) are benzyl adenine (BAP), indole-3-butyric acid (IBA), kinetin (KIN), and 1-naphthaleneacetic acid (NAA). Metal racks, cotton plugs, plastic covers, rubber bands, markers, glass jars (6 × 12 cm) ethanol spray bottles, a benzene burner, petri plates, and 70% ethanol.

### Methods

#### Preparation of culture media

The method used for preparation the media was followed that by Elsaid et al.^20^. To prepare one liter of Murashige and Skoog medium (MS), 600 ml of distilled water that was placed on a stirrer. Then 30 g of sucrose was added and swirled until it fully dissolved. Afterward 4.4 g of prepared MS medium was added and stirred till dissolved. The volume of the medium was completed using distilled water. The pH of the medium was set in the range of 5.6–5.8 using the pH meter, with drops of 10% Hydrochloric acid (HCl) or 1 M Sodium hydroxide (NaOH). The prepared culture medium was warmed, subsequently agar 0.8% (8 g/L) was added and stirred till a transparent solution was achieved. The culture medium was sterilized by autoclaving at 121 °C for 15 min; then, the hot medium was transferred into glass jars (50–60 mL/jar), left to cool, and sealed with autoclaved plastic lids. The jars were kept for 3 days at 23–25 °C, so as to ensure efficient sterilization. The explants for each treatment and repeated three times. They were subjected to a light cycle of 16 h of light and 8 h of darkness, with a light intensity of 3000 lx density provided by 40-Watt white cool fluorescent tubes with a maintaining 70 ± 5% relative humidity in the culture room.

#### Explant preparation and sterilization for callus induction

The explants were obtained by planting young stems and cutting them into uniform sizes (0.5–1 cm). After that they were washed four times under tape water. Then, they were submerged in 70% ethanol at various time intervals-0.5, 1, and 1.5-min and then immersed in sodium hypochlorite solution (5%) for different durations 1, 2, 3, and 4 min. Immersing the explant in ethanolic solution (70%) for 1 min and then in 5% sodium hypochlorite for 3 min was the best sterilization condition. The explant preparation and sterilization method followed Elsaid et al.^20^. Table 9 provides an overview of the results.

**Table 9:** Decontamination rate of different sterilization procedures on the explant of *S. Rebaudiana.*

Contact time with 70% ethanol (min)	Contact time with 5% hypochlorite (min)	Decontamination rate (%)
0.5	0.5	70
0.5	1	80
1	2	85
1	3	100
1.5	4	90

#### Induction of calli from the cultivated conventional plant

Cultivated seedlings from mature conventional plants were used as explants (stem) to produce Calli, representing the first stage of indirect micropropagation. Explants of uniform size (0.5–1 cm) were excised under sterile conditions. Then they were cultured in jars containing MS medium fortified by (3% *w/v*) sucrose and (8% *w/v*) agar, and pH was calibrated to 5.7. The MS medium was amended with diverse concentrations of PGRs, especially (1, 2, and 3 mg/L) Kin, NAA, and 1 mg/L of BAP. About fifteen jars were prepared, each containing ten explants. They were sub-cultured twice four-week intervals by transferring the developed callus to a fresh MS medium containing the same PGRs. The percentage of the fresh weight of the calli (in grams) and the induced calli was recorded. The method of callus induction was followed that Elsaid et al.^20^.

#### Shoot and root regeneration of *S. rebaudiana* via indirect- micropropagation

The induced eight-week-old calli were transferred to MS medium fortified with different concentrations of PGRs for shoot and root initiation. For shoot induction and multiplication, the medium was fortified with (0.5, 1, and 1.5 mg/L) BAP, while for root multiplication, the medium was fortified with 0.5, 1, and 2 mg/L IBA. After sixteen weeks of cultivation, the mean number of shoots and roots, the mean lengths of shoots and roots were calculated.

#### Explant Preparation and sterilization for direct micropropagation

Healthy apical and basal stem sprouts were collected from selected plants, and cuttings measuring 10–15 cm in length were taken. These cuttings are submerged in a 3.4 g/L solution of 5% sodium hypochlorite for one hour, followed by throughout rising with tap water for two hours. Subsequently, shoots measuring 1–3 cm in length, were selected as explants. The leaves were removed from the shoots and surface-sterilized by immersing them in 70% ethanol for 0.5–1 min, followed by submerging in a sodium hypochlorite solution containing 0.6% active chlorine and 2–3 drops of Tween 80 for 1, 2, and 3 min. The optimal sterilization condition was achieved by dipping explants in 1 min in 70% ethanol and immersing 3 min in a sodium hypochlorite solution containing 0.6% active chlorine and 2 to 3 drops of Tween 80. The plant preparation and sterilization methods are followed Elsaid et al.^20^. All results are shown in Table 10.

**Table 10:** Decontamination rate of different sterilization procedures on the basal and apical stem explant of *S. Rebaudiana.*

Contact time with 70% ethanol (min)	Contact time with 5% hypochlorite (addition 2 or 3 drops of Tween 80. (min)	Decontamination rate (%)
0.5	1	75
0.5	2	85
0.5	3	90
1	3	100

#### Shoot and roots regeneration of *S. rebaudiana* via direct-micropropagation

The sterilized basal and apical stem sprouts were cultured in different media in jars containing different concentrations of MS media (4.4, 3.3, and 2.2 g/L), sucrose (30 g/L), agar (8 g/L), and plant vitamins as myoinositol (10 and 15 mg/L), thiamine HCl (0.5, and 1 mg/L), pyridoxine-HCl (0.1 mg/L), and amended with varying level concentrations of elicitors 1.5, and 2 mg/L BAP, and 2 m/l IBA for shoot initiation, multiplication and root multiplication. After each stage -initiation, multiplication of shoot and root regeneration-the mean number of shoots and roots, the mean lengths of shoots and roots were recorded.

#### Extraction and yield analysis of soil-grown, callus, and regenerated *S. rebaudiana* through direct and indirect micropropagation

A total of 20 g from each group soil-grown, callus, and both direct and indirect micropropagation of *S. rebaudiana* were air-dried in the shade for two days. The samples were then macerated in 500 mL of 100% methanol four times until exhaustion, using a sonicator for 30 min during each run. The total methanolic extracts were concentrated under vacuum using a Rotavap (Hahin-shin, Japan) at 35 °C, to yield dry crude extracts from the aerial parts, calluses, and both direct and indirect micropropagated plants, resulting in 1.2 g, 100 mg, 370 mg, and 400 mg, respectively. These extracts were subjected to UPLC-MS analysis.

#### Methods for metabolite profiling using UPLC-ESI-MS/MS

Ultra-performance liquid chromatography-electrospray ionization tandem mass spectrometry (UPLC-ESI-MS/MS) was conducted for both qualitative identification and relative quantification of constituents in *S. rebaudiana* samples. The targeted secondary metabolites belonged to several classes of phytochemicals including, steviol glycosides, flavonoids and phenolic acids. The samples solution was prepared at concentration of (100 µg/mL) using HPLC- analytical grade methanol, then filtered through a 0.2µ membrane disc filter, before the analysis. On the injection, samples and standards 10 µL were injected into the UPLC instrument integrated with reverse-phase C-18 column (ACQUITY-UPLC-PDA detector—BEH-C18 (1.7 μm) particle size − 2.1 × 50 mm Column). The sample’s mobile phase was filtered through a 0.2 μm filter membrane disc and subsequently sonicated to remove any dissolved air before injected into the column. Elution of the mobile phase was accomplished at a flow rate 0.2 mL/min with a gradient mobile phase containing two eluents: Eluent A is consisted of acidified water (0.1% formic acid) and eluent B is consisted of acidified methanol (0.1% formic acid). The obtained spectra and peaks were analyzed with the help of the software (Maslynx 4.1) and the preliminary identification of all compounds was based on their retention time and mass spectrum compared to literature data. Metabolite quantification was achieved by determining relative peak areas according to the procedure that was previously mentioned in Elsaid et al.^20^.

#### Principal component analysis (PCA)

The data were analyzed using the SIMCA-P software package (version 13.0, Umetrics et al.) through principal component analysis (PCA).

#### Equipment for UPLC-ESI-MS/MS

The XEVO TQD triple quadrupole instrument mass spectrometer (Waters Corporation, Milford, MA01757, USA) was used in the ESI-MS positive ion acquisition mode. The chromatographic separation was performed using an ACQUITY UPLC BEH C_18_ Column, 130 Å, 1.7 μm, 2.1 mm X 50 mm, with a flow rate of 0.2 mL/min. The solvent system used contained water with 0.1% formic acid (A) and methanol with 0.1% formic acid (B).

### Statistical analysis

The statistical analysis was carried out using one-way-ANOVA with the Statistical Package for the Social Sciences (SPSS, Version 25). A post hoc Duncan’s test was applied to determine the significant differences at the (*p* ≤ 0.05) level.

## Data Availability

All data generated or analyzed during this study are included in this published article.

## References

[1] Abou-Hadid, A. F. Impact of climate change on Egyptian agriculture, challenges, and opportunities. *Clim. Changes Impacts Aquat. Environ. Assess. Adapt. Mitig Road. Map Sustain. Dev.* 171–182. 10.1007/978-3-031-74897-4_7 (2025).

[2] Unicef. Water scarcity in egypt: growing concerns, and partnerships. *Unicef Document* (2022). https://doi.org/https://www.unicef.org/egypt/documents/water-scarcity-egypt.

[3] Abdrabbo, M. A. A. et al. Climate change impact on economic and irrigation requirements for sugarcane crop in Egypt. (2021). 10.17170/kobra-202011192212.

[4] Report No. EG2024-0011, (USDA Foreign Agricultural Service, United States Departement of Agriculture. Egypt Sugar Annual Report & Washington, D. C. 2024). (2024). https://apps.fas.usda.gov/newgainapi/api/Report/DownloadReportByFileName?fileName=Sugar+Annual_Cairo_Egypt_EG2024-0011.pdf (accessed Jan 2025).

[5] Hamed, A. E., Salem, A. Z. M. & Abou El-Nour, H. M. Water footprint and productivity of stevia compared to sugar crops under Egyptian conditions. *Irrig. Drain. Syst. Eng.* **10**, 1–7. 10.4172/2168-9768.1000255 (2021).

[6] Borgo, J., Laurella, L. C., Martini, F., Catalán, C. A. & Sülsen, V. P. Stevia genus: phytochemistry and biological activities update. *Molecules*. **26**, 2733. 10.3390/molecules26092733 (2021). 34066562 10.3390/molecules26092733 PMC8125113

[7] Ahmad, J., Khan, I., Blundell, R., Azzopardi, J. & Mahomoodally, M. F. Stevia rebaudiana bertoni.: an updated review of its health benefits, industrial applications and safety. *Trends Food Sci. Technol.* **100**, 177–189. 10.1016/j.tifs.2020.04.030 (2020).

[8] Sharma, S. et al. Exploring plant tissue culture and steviol glycosides production in Stevia rebaudiana (Bert.) bertoni: A review. *Agriculture*. **13**, 475. 10.3390/agriculture13020475 (2023).

[9] Additives, E. P. et al. Safety evaluation of the food additive steviol glycosides, predominantly rebaudioside M, produced by fermentation using Yarrowia lipolytica VRM. *Efsa J.* **21**, e8387. 10.2903/j.efsa.2023.8387 (2023). 38125973 10.2903/j.efsa.2023.8387 PMC10731492

[10] Schiatti-Sisó, I. P., Quintana, S. E. & García-Zapateiro, L. A. Stevia (*Stevia rebaudiana*) as a common sugar substitute and its application in food matrices: an updated review. *J. Food Sci. Technol.* **60**, 1483–1492. 10.1007/s13197-022-05396-2 (2023). 37033318 10.1007/s13197-022-05396-2 PMC10076456

[11] Orellana-Paucar, A. M. Steviol glycosides from Stevia rebaudiana: an updated overview of their sweetening activity, Pharmacological properties, and safety aspects. *Molecules*. **28**, 1258. 10.3390/molecules28031258 (2023). 36770924 10.3390/molecules28031258 PMC9920402

[12] Papaefthimiou, M., Kontou, P. I., Bagos, P. G. & Braliou, G. G. Antioxidant activity of leaf extracts from Stevia rebaudiana Bertoni exerts attenuating effect on diseased experimental rats: A systematic review and meta-analysis. *Nutrients*. **15**, 3325. 10.3390/nu15153325 (2023). 37571265 10.3390/nu15153325 PMC10420666

[13] Prakash, I. & Chaturvedula, V. S. P. Steviol glycosides: natural noncaloric sweeteners. In *Reference Series in Phytochemistry* (eds. Merillon, J. M., Ramawat, K.) 101–128 (2018). 10.1007/978-3-319-26478-3_9-1.

[14] Ghonema, M. A. Biosafety of Stevia extract employing a variety of Short-Term genotoxic bioassays. *J. Adv. Agric. Res*. **19**, 722–737. 10.21608/jalexu.2014.160559 (2014).

[15] Ceunen, S. & Geuns, J. M. Steviol glycosides: chemical diversity, metabolism, and function. *J. Nat. Prod.* **76**, 1201–1228. 10.1021/np400203b (2013). 23713723 10.1021/np400203b

[16] Lemus-Mondaca, R., Vega-Gálvez, A., Zura-Bravo, L. & Ah-Hen, K. Stevia rebaudiana bertoni, source of a high-potency natural sweetener: A comprehensive review on the biochemical, nutritional and functional aspects. *Food Chem.* **132**, 1121–1132. 10.1016/j.foodchem.2011.11.140. (2012). 29243591 10.1016/j.foodchem.2011.11.140

[17] Tayel, D., Khamis, N. & Darwish, O. Artificial sweeteners consumption among Alexandria university students, Egypt. *J. High. Inst. Public. Health*. **47**, 1–7. 10.21608/jhiph.2017.19971 (2017).

[18] de Andrade, M. V. S., Lucho, S. R., de Castro, R. D. & Ribeiro, P. R. Alternative for natural sweeteners: improving the use of stevia as a source of steviol glycosides. *Ind. Crops Prod.* **208**, 117801. 10.1016/j.indcrop.2023.117801 (2024).

[19] Biswas, P., Kumari, A., Modi, A. & Kumar, N. Improvement and regulation of steviol glycoside biosynthesis in Stevia rebaudiana Bertoni. *Gene*. **891**, 147809. 10.1016/j.gene.2023.147809 (2024). 37722610 10.1016/j.gene.2023.147809

[20] Elsaid, M. B., Elnaggar, D. M., Owis, A. I., AbouZid, S. F. & Eldahmy, S. Production of isoquinoline alkaloids from the in vitro conserved Fumaria parviflora and their in vitro wound healing activity. *Nat. Prod. Res.* **36**, 2575–2579. 10.1080/14786419.2021.1904401 (2022). 33823691 10.1080/14786419.2021.1904401

[21] Long, Y., Yang, Y., Pan, G. & Shen, Y. New insights into tissue culture plant-regeneration mechanisms. *Front. Plant Sci.* **13**, 926752. 10.3389/fpls.2022.926752 (2022). 35845646 10.3389/fpls.2022.926752 PMC9280033

[22] Miladinova-Georgieva, K. et al. Effects of different elicitors on micropropagation, biomass and secondary metabolite production of *Stevia rebaudiana* Bertoni—a review. *Plants *. **12**(1), 153. 10.3390/plants12010153 (2023). 10.3390/plants12010153 PMC9824860 36616282

[23] Owis, A., Abdelwahab, N. & Abul-Soad, A. Elicitation of phenolics from the micropropagated endangered medicinal plant *Calligonum polygonoides* L. (Polygonoaceae). *Pharmacogn. Mag.* **12**(47): 465 (2016). 10.4103/0973-1296.191458 PMC5068125 27761076

[24] Fayezizadeh, M. R., Ansari, N. A., Sourestani, M. M. & Hasanuzzaman, M. Variations in photoperiods and their impact on yield, photosynthesis and secondary metabolite production in Basil microgreens. *BMC Plant Biol.* **24**, 712. 10.1186/s12870-024-05448-z (2024). 39060976 10.1186/s12870-024-05448-z PMC11282849

[25] Rodriguéz-Páez, L. A. et al. Micropropagation protocols for three elite genotypes of Stevia rebaudiana Bertoni. *Horticulturae*. **10**, 404. 10.3390/horticulturae10040404 (2024).

[26] Dyduch-Siemińska, M., Wawerska, K. & Gawroński, J. The potential of plant tissue cultures to improve the steviol glycoside profile of Stevia (Stevia rebaudiana Bertoni) regenerants. *Int. J. Mol. Sci.* **25**, 13584. 10.3390/ijms252413584 (2024). 39769347 10.3390/ijms252413584 PMC11677599

[27] Ghazal, B. et al. Elicitors directed in vitro growth and production of stevioside and other secondary metabolites in Stevia rebaudiana (Bertoni) Bertoni. *Sci. Rep.* **14**, 14714. 10.1038/s41598-024-65483-6 (2024). 38926419 10.1038/s41598-024-65483-6 PMC11208548

[28] Madhu, G., Sathyanarayana, B. N. & Kumar, R. V. Genotypic variability in in vitro propagation of Stevia rebaudiana. *J. Appl. Res. Med. Aromatic Plants*. **17**10.1016/j.jarmap.2020.100255 (2020).

[29] Masoumi, B., Tabibiazar, M., Golchinfar, Z., Mohammadifar, M. & Hamishehkar, H. A review of protein-phenolic acid interaction: reaction mechanisms and applications. *Crit. Rev. Food Sci. Nutr.* **64**, 3539–3555. 10.1080/10408398.2022.2132376 (2024). 36222353 10.1080/10408398.2022.2132376

[30] Yu, H. et al. Effect of *Stevia rebaudiana* stem waste extract on lipid oxidation of salted-dried Pacific Saury during chilled storage. *LWT* **200**, 116180. 10.1016/j.lwt.2024.116180 (2024).

[31] Wang, F., Kong, B. L. H., Tang, Y. S., Lee, H. K. & Shaw, P. C. Bioassay guided isolation of caffeoylquinic acids from the leaves of Ilex pubescens hook. Et arn. And investigation of their anti-influenza mechanism. *J. Ethnopharmacol.* **309**, 116322. 10.1016/j.jep.2023.116322 (2023). 36868436 10.1016/j.jep.2023.116322

[32] Fu, Y. et al. Characterization and quantification of phenolic constituents in Peach blossom by UPLC-LTQ-orbitrap-MS and UPLC-DAD. *Nat. Prod. Commun.* **15**10.1177/1934578X19884437 (2020).

[33] Bouymajane, A. et al. Phenolic compound, antioxidant, antibacterial, and in Silico studies of extracts from the aerial parts of *Lactuca saligna* L. *Molecules*. **29**, 596. 10.3390/molecules29030596 (2024). 38338341 10.3390/molecules29030596 PMC10856452

[34] Gao, H. et al. Isolation, structure Elucidation and neuroprotective effects of caffeoylquinic acid derivatives from the roots of *Arctium lappa* L. *Phytochemistry*. **177**, 112432. 10.1016/j.phytochem.2020.112432 (2020). 32562918 10.1016/j.phytochem.2020.112432

[35] Nguyen, H. C. et al. Bioactive compounds, antioxidants, and health benefits of sweet potato leaves. *Molecules*. **26**, 1820. 10.1016/j.focha.2024.100693 (2021). 33804903 10.3390/molecules26071820 PMC8038024

[36] Olennikov, D., Chirikova, N. & Tsyrenzhapov, A. Phenylpropanoids from Parasenecio hastatus (Compositae) and their wound-healing activity. *Russ. J. Bioorg. Chem.* **47**, 1411–1417. 10.1134/S106816202107013X (2021).

[37] Makori, S. I., Mu, T. H. & Sun, H. N. Physicochemical properties, antioxidant activities, and binding behavior of 3, 5-di-O-caffeoylquinic acid with beta-lactoglobulin colloidal particles. *Food Chem.* **347**, 129084. 10.1016/j.foodchem.2021.129084 (2021). 33486366 10.1016/j.foodchem.2021.129084

[38] Islam, S., Adam, Z. & Akanda, J. H. Quinic and caffeic acids derivatives: affecting antioxidant capacities and phenolics contents of certain therapeutic and specialty crops employing water and ethanolic extracts. *Food Chem. Adv.* **4**, 100693. 10.1016/j.focha.2024.100693 (2024).

[39] Ning, Q. et al. 3-O-Caffeoylquinic acid in Periploca Forrestii schltr extract ameliorates collagen-induced arthritis by inducing IL17/IL23 cells in rats. *Trop. J. Pharm. Res.* **21**, 1445–1452. 10.4314/tjpr.v21i7.13 (2022).

[40] Liu, C. et al. Chemical profiling of Kaliziri injection and quantification of six caffeoyl Quinic acids in beagle plasma by LC-MS/MS. *Pharmaceuticals*. **15**, 663. 10.3390/ph15060663 (2022). 35745582 10.3390/ph15060663 PMC9230828

[41] Lee, D. et al. Insulin secretion and α-glucosidase inhibitory effects of dicaffeoylquinic acid derivatives. *Appl. Biol. Chem.* **65**, 22. 10.1186/s13765-022-00688-9 (2022). https://doi.org/.

[42] Han, S. Y., Kim, J., Kim, B. K., Whang, W. K. & Min, H. Effects of caffeoylquinic acid analogs derived from aerial parts of Artemisia Iwayomogi on adipogenesis. *Food Sci. Biotechnol.* **32**, 1215–1223. 10.1007/s10068-023-01262-9 (2023). 37362808 10.1007/s10068-023-01262-9 PMC10289966

[43] Zhao, Y., Ren, Y., Liu, Z., Wang, Z. & Liu, Y. The metabolite profiling of 3, 4-dicaffeoylquinic acid in Sprague–Dawley rats using ultra‐high performance liquid chromatography equipped with linear ion trap‐Orbitrap MS. *Biomed. Chromatogr.* **36**, e5276. 10.1002/bmc.5276 (2022). 34741336 10.1002/bmc.5276

[44] Simoens, C. et al. Pharmacokinetics of oral rebaudioside A in patients with type 2 diabetes mellitus and its effects on glucose homeostasis: A Placebo-Controlled crossover trial. *Eur. J. Drug Metab. Pharmacokinet.* **47**, 827–839. 10.1007/s13318-022-00792-7 (2022). 36057030 10.1007/s13318-022-00792-7 PMC9440320

[45] Gupta, A., Atanasov, A. G., Li, Y., Kumar, N. & Bishayee, A. Chlorogenic acid for cancer prevention and therapy: current status on efficacy and mechanisms of action. *Pharmacol. Res.* **186**, 106505. 10.1016/j.phrs.2022.106505 (2022). 36243332 10.1016/j.phrs.2022.106505

[46] Tian, W. et al. Caffeic acid and chlorogenic acid mediate the ADPN-AMPK-PPARα pathway to improve fatty liver and production performance in laying hens. *J. Anim. Sci. Biotechnol.* **16**, 49. 10.1186/s40104-025-01175-z (2025). 40176148 10.1186/s40104-025-01175-z PMC11966898

[47] Sun, L., Tao, S. & Zhang, S. Characterization and quantification of polyphenols and triterpenoids in thinned young fruits of ten Pear varieties by UPLC-Q TRAP-MS/MS. *Molecules* **24**, 159. 10.3390/molecules24010159 (2019). 30609827 10.3390/molecules24010159 PMC6337724

[48] Mateos, R., Baeza, G., Sarriá, B. & Bravo, L. Improved LC-MSn characterization of hydroxycinnamic acid derivatives and flavonols in different commercial mate (Ilex paraguariensis) brands. Quantification of polyphenols, methylxanthines, and antioxidant activity. *Food Chem.* **241**, 232–241. 10.1016/j.foodchem.2017.08.085 (2018). 28958524 10.1016/j.foodchem.2017.08.085

[49] Ao, X. et al. Extraction, isolation and identification of four phenolic compounds from Pleioblastus Amarus shoots and their antioxidant and anti-inflammatory properties in vitro. *Food Chem.* **374**, 131743. 10.1016/j.foodchem.2021.131743 (2022). 34915365 10.1016/j.foodchem.2021.131743

[50] Hao, B., Yang, Z., Liu, H., Liu, Y. & Wang, S. Advances in flavonoid research: sources, biological activities, and developmental prospectives. *Curr. Issues. Mol. Biol.* **46**, 2884–2925. 10.3390/cimb46040181 (2024). 38666911 10.3390/cimb46040181 PMC11049524

[51] Pacifico, S. et al. New insights into phenol and polyphenol composition of Stevia rebaudiana leaves. *J. Pharm. Biomed. Anal.* **163**, 45–57. 10.1016/j.jpba.2018.09.046 (2019). https://doi.org/. 30286435 10.1016/j.jpba.2018.09.046

[52] Li, C. & Seeram, N. P. Ultra-fast liquid chromatography coupled with electrospray ionization time‐of‐flight mass spectrometry for the rapid phenolic profiling of red maple (Acer rubrum) leaves. *J. Sep. Sci.* **41**, 2331–2346. 10.1002/jssc.201800037 (2018). 29512337 10.1002/jssc.201800037 PMC7167591

[53] Thomasi, R. M. et al. Antiviral activity of flavonoids from Bauhinia holophylla leaves against Zika virus. *Microbiol. Res.* **15**, 582–597. 10.3390/microbiolres15020038 (2024).

[54] Lee, S. Y. & Shaari, K. LC–MS metabolomics analysis of *Stevia rebaudiana* Bertoni leaves cultivated in Malaysia in relation to different developmental stages. *Phytochem. Anal.* **33**, 249–261. 10.1002/pca.3084 (2022). 34490671 10.1002/pca.3084

[55] Huang, W. et al. Rational design for the complete synthesis of stevioside in Saccharomyces cerevisiae. *Microorganisms*. **12**, 1125. 10.3390/microorganisms12061125 (2024). 38930507 10.3390/microorganisms12061125 PMC11206123

[56] Phungsiangdee, Y., Chaothong, P., Karnpanit, W. & Tanaviyutpakdee, P. Validation of UHPLC-ESI-MS/MS method for determining steviol glycoside and its derivatives in foods and beverages. *Foods* **12**, 3941. 10.3390/foods12213941 (2023). 37959060 10.3390/foods12213941 PMC10647612

[57] Zhang, C. et al. Stevioside Ameliorates Palmitic Acid–Induced Abnormal Glucose Uptake via the PDK4/AMPK/TBC1D1 Pathway in C2C12 Myotubes. *Endocrinol. Diabetes Metab.* **7**, e00482. 10.1002/edm2.482 (2024). 10.1002/edm2.482 PMC10982459 38556697

[58] Ferdous, J., Bhuia, M. S., Chowdhury, R., Sheikh, S. & Islam, M. T. Therapeutic effects of natural food additives steviol glycosides from *Stevia rebaudiana*: A comprehensive review with mechanisms. *J. Food Biochem.* **2025**, 7772203 (2025). 10.1155/jfbc/7772203 (2025).

[59] Casas-Grajales, S. et al. Antioxidant and Immunomodulatory activity induced by stevioside in liver damage: in vivo, in vitro and in silico assays. *Life Sci.* **224**, 187–196. 10.1016/j.lfs.2019.03.035 (2019). 30890404 10.1016/j.lfs.2019.03.035 PMC6556435

[60] Talevi, A. Potential medicinal effects and applications of stevia constituents. *Phytochem. Rev.* **21**, 161–178. 10.1007/s11101-021-09753-5( (2022).

[61] Xu, Q. et al. Stevioside improves antioxidant capacity and intestinal barrier function while attenuating inflammation and apoptosis by regulating the NF-κB/MAPK pathways in diquat-induced oxidative stress of IPEC-J2 cells. *Antioxidants*. **12**, 1070. 10.3390/antiox12051070 (2023). 37237936 10.3390/antiox12051070 PMC10215602

[62] Gelinas, B. S., Liu, Y., Tello, E. & Peterson, D. G. Untargeted LC-MS based identification of rebaudioside A degradation products impacting flavor perception during storage. *Food Chem.* **373**, 131457. 10.1016/j.foodchem.2021.131457 (2022). 34736072 10.1016/j.foodchem.2021.131457

[63] Huang, C. et al. Properties, extraction and purification technologies of Stevia rebaudiana steviol glycosides: A review. *Food Chem.* 139622. 10.1016/j.foodchem.2024.139622 (2024). 10.1016/j.foodchem.2024.139622 38761729

[64] Xi, D. et al. Rebaudioside affords hepatoprotection ameliorating sugar sweetened beverage-induced nonalcoholic steatohepatitis. *Sci. Rep.* **10**, 6689 (2020). 32317687 10.1038/s41598-020-63688-z PMC7174355

[65] Li, P., Wang, Z., Lam, S. M. & Shui, G. Rebaudioside A enhances resistance to oxidative stress and extends lifespan and healthspan in *Caenorhabditis elegans*. *Antioxidants* **10**, 262. 10.3390/antiox10020262 (2021). 33567712 10.3390/antiox10020262 PMC7915623

[66] Ganter, J., Hellwig, E., Doerken, S. & Al-Ahmad, A. *Vitro* evaluation of the cariogenic potential of rebaudioside A compared to sucrose and xylitol. *Clin. Oral Investig.* **24**, 113–122. 10.1007/s00784-019-02908-x (2020). 31030274 10.1007/s00784-019-02908-x

[67] Yang, Z., Uhler, B., Zheng, T. & Adams, K. M. Enzymatic synthesis and characterization of a novel α-1→ 6-Glucosyl rebaudioside C derivative sweetener. *Biomolecules*. **9**, 27. 10.3390/biom9010027 (2019). 30646526 10.3390/biom9010027 PMC6358748

[68] Gonçalves Nunes, W. D., Russo, M., da Silva Bolzani, H., Caires, F. J. & V. & Thermal characterization and compounds identification of commercial Stevia rebaudiana Bertoni sweeteners and thermal degradation products at high temperatures by TG–DSC, IR and LC–MS/MS. *J. Therm. Anal. Calorim.* **146**, 1149–1155. 10.1007/s10973-020-10104-3 (2021).

[69] Dyduch-Siemińska, M. et al. Stevia rebaudiana bertoni, a source of high-potency natural sweetener—biochemical and genetic characterization. *Molecules*. **25**, 767. 10.3390/molecules25040767 (2020). 32053920 10.3390/molecules25040767 PMC7070548

[70] Ameer, K., Jiang, G. H., Amir, R. M. & Eun, J. B. In *Pathology*, 345–356 (Elsevier, 2020).

[71] Singh, S. & Rao, G. Stevia: The herbal sugar of 21st century. *Sugar Tech.* **7**, 17–24. 10.1007/BF02942413 (2005).

[72] De Santis, A., Sannicandro, K., Bellini, C. & Minerva, T. Trends in the use of multivariate analysis in educational research: a review of methods and applications in 2018–2022. *Je-lks J. e-learning Knowl. Soc.* **20**, 47–55. 10.20368/1971-8829/1135946 (2024).

